# Bioprocessing of Barley and Lentil Grains to Obtain In Situ Synthesis of Exopolysaccharides and Composite Wheat Bread with Improved Texture and Health Properties

**DOI:** 10.3390/foods10071489

**Published:** 2021-06-27

**Authors:** Giuseppe Perri, Carlo Giuseppe Rizzello, Marco Ampollini, Giuseppe Celano, Rossana Coda, Marco Gobbetti, Maria De Angelis, Maria Calasso

**Affiliations:** 1 Department of Soil, Plant and Food Science, University of Bari Aldo Moro, 70126 Bari, Italy; giuseppe.perri483@virgilio.it (G.P.); giuseppe.celano@uniba.it (G.C.); maria.deangelis@uniba.it (M.D.A.); 2 Department of Environmental Biology, University of Roma La Sapienza, 00185 Roma, Italy; carlogiuseppe.rizzello@uniroma1.it; 3 Puratos Italia S.r.l., 43122 Parma, Italy; mampollinI@puratos.com; 4 Department of Food and Nutrition, University of Helsinki, 00100 Helsinki, Finland; rossana.coda@helsinki.fi; 5 Helsinki Institute of Sustainability Science, Department of Food and Nutrition, University of Helsinki, 00100 Helsinki, Finland; 6 Faculty of Science and Technology, Free University of Bozen, 39100 Bozen, Italy; marco.gobbetti@unibz.it

**Keywords:** barley, bioprocessing, baked goods, dextran, fibers, germination, glycemic index, lactic acid bacteria, lentil, sourdough

## Abstract

A comprehensive study into the potential of bioprocessing techniques (sprouting and sourdough fermentation) for improving the technological and nutritional properties of wheat breads produced using barley and lentil grains was undertaken. Dextran biosynthesis in situ during fermentation of native or sprouted barley flour (B or SB) alone or by mixing SB flour with native or sprouted lentil flour (SB-L or SB-SL) by *Weissella paramesenteroides* SLA5, *Weissella confusa* SLA4, *Leuconostoc pseudomesenteroides* DSM 20193 or *Weissella confusa* DSM 20194 was assessed. The acidification and the viscosity increase during 24 h of fermentation with and without 16% sucrose (on flour weight), to promote the dextran synthesis, were followed. After the selection of the fermentation parameters, the bioprocessing was carried out by using *Leuconostoc pseudomesenteroides* DSM 20193 (the best LAB dextran producer, up to 2.7% of flour weight) and a mixture of SB-SL (30:70% *w*/*w*) grains, enabling also the decrease in the raffinose family oligosaccharides. Then, the SB-SL sourdoughs containing dextran or control were mixed with the wheat flour (30% of the final dough) and leavened with baker’s yeast before baking. The use of dextran-containing sourdough allowed the production of bread with structural improvements, compared to the control sourdough bread. Compared to a baker’s yeast bread, it also markedly reduced the predicted glycemic index, increased the soluble (1.26% of dry matter) and total fibers (3.76% of dry matter) content, giving peculiar and appreciable sensory attributes.

## 1. Introduction

Bioprocessing of cereals and legumes by using microbial inoculants, with and without the use of commercial enzymes, along with lactic acid bacteria (LAB) fermentation, are performed to improve their technological and functional properties, nutritional value, and consumer acceptability [1,2,3,4,5]. As one of the oldest and natural biotechnologies, sourdough fermentation by LAB offers multiple benefits for bread producers and consumers enhancing the overall quality of baked goods [3,6]. The pro-health effects of bioprocessing for making traditional and novel baked goods includes the microbial metabolites as well as the ability to affecting the levels and the bioavailability of several bioactive compounds, the capability to degrade anti-nutritional factors, the improvement of protein digestibility and the reduction of the glycemic index [6,7,8,9,10]. Among the well-known benefits of LAB fermentation on baked-food properties [11,12], some LAB strains have the ability to synthesize exopolysaccharides (EPS) [13,14,15]. EPS are polysaccharides commercially used in the food industry as emulsifiers, stabilizers, thickeners, gelling agents, as well as for moisture retention [16,17]. In the last decades, the use of EPS-producing starters has received increasing interest from the bakery and cereal global industry since the hydrocolloidal nature of these carbohydrate polymers provides a natural replacement for commercial ones such as hydroxypropyl methylcellulose (HPMC) [18,19]. For several EPS, prebiotic effects have also been described [20]. Moreover, for EPS has been reported additional effects, such as anti-inflammatory, antitumor, or antioxidant properties [21]. Among the EPS, the homopolysaccharides dextran and levan are those of relevance for the bakery industry [18,19,22].

α-D-glucans (dextran, mutan, alternan, and reuteran) are homopolysaccharides produced by extracellular glucansucrases, using sucrose as the substrate [23]. Dextran can be produced in situ in fermented products by LAB (e.g., *Leuconostoc* and *Weissella* spp.) or acetic acid bacteria and it has been generally recognized as safe (GRAS) by the Food and Drug Administration. Dextran has been successfully produced in cereals, pseudocereals and legumes by fermentation with selected LAB strains to obtain breads with an enhanced quality and prolonged shelf-life [24,25,26,27,28].

Nowadays, several studies have established the use of germinated grains as means to innovate and obtain foods with improved nutritional quality and content of bioactive compounds [29,30,31,32]. The potential of grain germination as an effective, low-cost and sustainable practice to enhance the levels of functional compounds and healthy properties, but also the digestibility, bioavailability and palatability of grains, has been highlighted before (for review see Benincasa et al. 2019) [31]. 

Germinated grains can be considered and labelled as malted or sprouted whole grain because of containing all the original bran, germ, and endosperm [31]. In the last decade, several composite breads containing sprouted grains have been developed and beyond the wheat [30], minor cereals such as barley [33], pseudocereals [33], and legumes [34,35] have been investigated as germinated ingredients for making functional bread. Although the addition of flour from sprouted grains to wheat bread improves its nutritional value, some detrimental effects on bread rheology and flavor can be observed [6,35], with respect to the conventional counterpart. To overcome this technological drawback, EPS can be used since they have been proven to be good texture modifiers [36,37].

In our previous study [35], we have demonstrated that the addition of 30% *w*/*w* sourdoughs from lentil and sprouted lentil flours enriched with bacterial dextran in white bread increased the fiber content, specific volume and decreased crumb hardness and staling rate compared to wheat control bread, without negative effects on its sensory characteristics.

In this study, we aimed to assess the potential of the bioprocessing of barley (*Hordeum vulgare*) and lentil (*Lens culinaris*) flours by lactic acid bacteria to obtain a sourdough enriched with dextran useful to maximize the texture and health-promoting properties of composite barley–lentil–wheat breads. The main reasons for utilization of barley and lentil grains in food are the health benefits deriving from their peculiar nutritional composition, resulting in wanted functional attributes and improved bread quality. In particular, studies have shown dietary fibers to have many health benefits. Barley is an excellent source of dietary fibers, especially β-glucan, known to have several physiological functions, including the improvement of lipid metabolism and the increase in satiety [38,39,40,41]. Lentils is a legume commonly consumed worldwide, particularly in the Mediterranean area, which have high protein content and low caloric value, contain phytochemicals and present antioxidant properties [42]. Despite their large use as food ingredients, the nutritional quality of lentils and derived products may be decreased by the content of ANFs [6]. Germination (sprouting) and fermentation of barley and lentil flours with the simultaneous in situ production of EPS are a potential opportunity to reduce the quality losses. The positive effect of sprouted barley and sprouted lentil flours as a nutritious and functional ingredient has been shown in previous studies [32,34,43].

To this aim, the suitability of native or sprouted barley alone or a blend of sprouted barley with native or sprouted lentil grains as a substrate for dextran synthesis by *Weissella paramesenteroides* SLA5, *Weissella confusa* SLA4, *Leuconostoc pseudomesenteroides* DSM 20193 or *Weissella confusa* DSM 20194, previously shown as good dextran producers [26,27,35,36,44], was assessed. The acidification and the viscosity increase during 24 h of fermentation with and without added sucrose were followed. The best sourdoughs were characterized and used for the manufacture of laboratory-scale composite wheat breads. The breads were evaluated for their rheological, nutritional and sensory properties. 

## 2. Materials and Methods

### 2.1. Materials

The ingredients used in this study included barley grains (*Hordeum vulgare*, Caporal Grani s.a.s.) (carbohydrate 78.8% on dry matter (on d.m.), fibers 13.5% on d.m., protein (N × 5.70) 13.6% on d.m., fat 1.5% on d.m., moisture 11.1%), lentil grains (*Lens culinaris*, Caporal Grani s.a.s.) (carbohydrate 50.4% on d.m., protein 30.0% on d.m., fat 0.66% on d.m., fibers 23% on d.m., moisture 11%), wheat flour (*Triticum aestivum*, commercial wheat flour type “0”, Puratos Italia s.a.s., protein 13% on d.m., fat 1.9% on d.m., fiber 2.3% on d.m., moisture 13.6%), fresh yeast (Puratos Italia), sucrose (Sigma Aldrich, St. Louis, MO, USA) and salt. Barley and lentil grains were sprouted according to the protocol described by Montemurro et al. [33]. Briefly, whole grains were prior disinfected by submersion in 1.25% *w/v* NaClO (seed:water ratio 1:5 *w*/*v*) for 30 min at room temperature, washed 20–30 min under tap water and then soaked in water at 16.5 °C for 24 h. Afterwards, grains were placed in a germination system (BioSnacky, Biokosma GmbH, Konstanz, Germany) in the dark until they began to sprout (rootlets length correspondent to ca. ¾ of the seed length). After germination, sprouted grains, including the rootlets which were not separated, were washed with distilled water and dried in experimental conditions comparable to those used for industrial malting of barley [33]. Flours were obtained from native and sprouted grains by a laboratory mill (IKA-Werke M20 GMBH, and Co. KG, Staufen, Germany). After milling, all the flours were sieved (mesh size 500 μm) to remove the coarse fraction and stored under vacuum until further analysis. 

### 2.2. Liquid Sourdough Fermentation

*Weissella paramesenteroides* SLA5 and *Weissella confusa* SLA4, belonging to the Culture Collection of the Department of Soil, Plant and Food Sciences (University of Bari Aldo Moro, Italy) previously isolated from sprouted lentil flour [45], *Leuconostoc pseudomesenteroides* DSM 20193 and *Weissella confusa* DSM 20194 from DSMZ-German Collection of Microorganisms and Cell Cultures GmbH (Braunschweig, Germany), were used in this study as starters for preliminary dough fermentation. The strains were selected based on their confirmed dextran-producing capacity using 58.4 mM sucrose as a carbon source [26,27,35,44] and pro-technological properties and sensory characteristics on native or sprouted lentil-based substrates [35]. *Lactiplantibacillus plantarum* (formerly *Lactobacillus plantarum*) DPPMAB24W (culture collection of DiSSPA, University of Bari), was used as a non-EPS-producing control [44]. All LAB strains were maintained in 20% (*v*/*v*) glycerol as frozen stocks at −20 °C and routinely propagated in de Man Rogosa and Sharpe (MRS) broth at 30 °C (Oxoid, Basingstoke, Hampshire, England). 

Selected strains were used as a single starter for fermentation of preliminary formulations of liquid doughs obtained by native or sprouted barley flour (B and SB, respectively) alone or by mixing SB flour with native or sprouted lentil flour (L or SL, respectively) in 40:60 and 30:70 ratios. The tested dough yield (DY, dough weight × 100/flour weight) were 500 for barley or sprouted barley dough alone and 333, 350, 400, 450 or 500 for the blends (Table S1). To support in situ formation of EPS, sucrose-supplemented (5% on dough weight, corresponding to 16% flour weight, f.w.) fermentations were carried out (namely EPS-positive sourdoughs, EPS POS) [36]. For each condition, an EPS-negative sourdough (namely EPS NEG) was prepared with the same starter strain but without sucrose addition [46]. For each formulation, a control dough (CT) without sucrose and without inoculum and a non-EPS-producing control dough (named B24W) added of sucrose and started by *L. plantarum* DPPMAB24W were prepared as described above [44]. Fermentations were carried out at 20 and 25 °C for 24 h. All the doughs were prepared in triplicate in sterile beakers using tap water and mixed manually for 5 min. Before and immediately after fermentation, samples were collected, stored at 4 °C and analyzed within 2 h. All the analyses were carried out in duplicate for each batch of sourdough (a total of six analyses for each type of sourdough). When used for sourdough fermentation, LAB cells were cultivated in MRS broth supplemented with 58.4 mM sucrose overnight, centrifuged (10,000× *g* for 10 min), washed in 50 mM phosphate buffer pH 7.0 (twice), and re-suspended in the water used for making the dough at the initial cell density of ca. 7 log cfu/g [44].

### 2.3. pH and Viscosity Measurement

The pH values of the sourdoughs were measured using a food pH meter equipped with a penetration probe (Model HI-99161, Hanna Instruments, Woonsocket, RI, USA). Viscosity of sourdoughs was measured before and after fermentation at 20 °C with a RheolabQC rheometer (Anton Paar, Graz, Austria), using 60 g of each dough mixed thoroughly. Viscosity was performed under different shear rates, from 2 to 100 1/s (up and down sweeps) [44] and the viscosity values at the shear rate of 100 1/s were compared. Based on pH and viscosity [27], the DY of 333, the temperature of 20 °C, the SB-L and SB-SL flour blends at a 30:70 ratio, were chosen to produce sourdoughs fermented by *L. pseudomesenteroides* DSM 20193, which were further characterized.

### 2.4. Enumeration of Cultivable Bacteria and Yeasts

Enumeration of cultivable bacteria and yeasts was carried out according to methods previously described [47]. For each dough, aliquots of 20 g were added to 180 mL of sterile sodium chloride solution (0.9%, *w*/*v*), homogenized with a Stomacher for 180 sec and serially diluted. Appropriate dilutions were plated in selective culture media and supplements purchased from Oxoid (Basingstoke, Hampshire, United Kingdom). Total mesophilic aerobic microorganisms were enumerated using Plate Count Agar (PCA) media after incubating at 30 °C for 48 h under aerobic condition. LAB were estimated using modified MRS, containing 28 mM maltose, 5%, *v/v* fresh yeast extract, pH 5.6, and supplemented with cycloheximide (0.1 g/L), incubating the plates under anaerobiosis (AnaeroJar and AnaeroGen, Oxoid) at 30 °C for 48 h. *Enterobacteriaceae* were enumerated using a Violet Red Bile Glucose Agar (VRBGA) medium and plates were incubated at 37 °C for 24 h. Yeast cells were enumerated by using Wort agar supplemented with chloramphenicol (0.1 g/L), incubating the plates at 30 °C for 48 h. To confirm the microbiological counts, representative colonies from each medium were analyzed for morphology, motility, Gram staining reaction and catalase test.

### 2.5. Determination of Dextran, Sugars and Organic Acids

Before and after fermentation, the amount of dextran was determined by an enzyme-assisted method based on the enzymatic activity of the dextranase from *Chaetomium erraticum* (10,000 nkat/g) (Sigma-Aldrich, Darmstadt, Germany) and α-glucosidase from *Aspergillus niger* (1000 kat/g) (Megazyme, Ireland), as previously described by Katina et al. [37]. After freeze-drying, removal of free sugars and short oligosaccharides and inactivation of the enzymes, glucose in the sourdough supernatants was analyzed by high performance anion exchange chromatography with pulsed amperometric detection (HPAEC-PAD), using glucose (Merck, Darmstadt, Germany) as standard and 2-deoxy-D-galactose (Sigma-Aldrich, Dorset, UK) as the internal standard for quantification [37]. Results were calculated as the sum of anhydro-glucose using a corrector factor of 0.90.

For sugar analysis, freeze-dried sourdough before and after fermentation was treated to inactivate enzymes and microbes [35,44] and to remove any polymeric molecules. The samples were analyzed by HPAEC-PAD as reported by Xu et al. [43]. Results are expressed as % on a flour weight basis. 

The total titratable acidity (TTA) was measured on 10 g of dough diluted with 90 mL of sterile sodium chloride (0.9% *w*/*v*) solution titrated with 0.1 mol/L NaOH until pH achieved 8.3. TTA was expressed as the total NaOH amount (mL). 

Organic acid analysis (lactic acid and acetic acid contents) in the extracts from sourdoughs before and after fermentation was performed using commercial kits, K-DLATE and K-ACET (Megazyme, Wicklow, Ireland) kits. The quotient of fermentation (FQ) was calculated as the molar ratio between lactic and acetic acids. 

### 2.6. Bread Making Trials

Experimental breads were manufactured at the pilot plant of Puratos Italia (Ceparana, La Spezia, Italy). Three types of breads were prepared: control wheat bread manufactured using wheat flour fermented by baker’s yeast alone (CWB); sourdough wheat bread (SWB) manufactured using sprouted barley–sprouted lentil sourdough (SB-SL SWB EPS NEG); sourdough wheat bread manufactured using dextran-containing sprouted barley–sprouted lentil sourdough (SB-SL SWB EPS POS). Selected sourdoughs were used in baking at 30% of the dough weight corresponding to a 15% of wheat flour substitution. The selection of the percentage of replacement was based on a calculation (nutritional composition) to obtain a 3% dietary fibers content which allows the nutrition claim “source of fibers” [48].

The required amount of water for the breads was previously determined by a Brabender farinograph (Brabender GmbH & Co. KG, Duisburg, Germany). The amount of flour and water was the same in CWB and SWB breads (DY 162). All breads were manufactured according to a two-stage protocol, which is routinely used in artisanal and industrial bakeries [47]. In stage I, the SB-SL blended flours obtained from sprouted barley and lentil grains were fermented with *L. pseudomesenteroides* DSM 20193 for 24 h at 20 °C in sucrose-supplemented or not fermentation as described before. In stage II, SB-SL EPS NEG or EPS POS sourdoughs were mixed with all ingredients (wheat flour, water and baker’s yeast) in a mixer vessel (Sottoriva S.p.a Group). Baker’s yeast was added at a percentage of 1.1% *w*/*w*. The doughs were divided into pieces of uniform weight (500 g), rested in pans for 20 min at 25 °C and relative humidity (RH) of 75% and leavened in a fermentation chamber (Zucchelli Forni S.p.a) for 60 min at 30 °C and RH 85%. All types of breads were baked at 220 °C for 30 min in a rotating rack oven (Zucchelli Forni S.p.a). Five replicates for each type of bread were carried out on two different days. All the analyses were carried out in duplicate for three replicates of bread (a total of six analyses for each type of bread). The resulting breads were allowed to cool to room temperature (25 °C) for 2 h before analyses and weighing. 

### 2.7. Bread Technological Characterization

Dextran-containing SWB (EPS POS) were compared to EPS-negative SWB (EPS NEG) and to the control breads [36]. After cooling, loaves were weighed, and loaf volume measured by millet-seed displacement [49]. The specific volume of the bread was calculated by dividing the loaf volume (mL) by the corresponding loaf weight (g). The percent bake loss of the breads was also evaluated (% bake loss = (dough weight−bread weight) * 100/dough weight). Bread was packed in polypropylene micro perforated bags and stored for 7 days at room temperature. For each bread, slices (2 cm) were used for texture profile analysis (TPA) after days 1 and 7 of storage at room temperature using a texture analyzer (TA, TA-XT2i, Stable Micro Systems Ltd., UK) fitted with a cylinder probe (diameter 36 mm) [50]. Data were recorded using a TPA analyzer Stable Micro Systems software exponent (version 5.0.9.0). All measurements were performed in triplicate by two compression cycles, test speed of 1 mm/s, and 30% compression [51]. The texture properties determined were: hardness (maximum peak force), springiness (ratio of a product’s original height), cohesiveness (the area of work during the second compression divided by the area of work during the first compression) and resilience (ratio of the first decompression area to the first compression area). The effect of sourdough on the staling rate was studied as the increase in hardness (staling rate = (hardness [day 7−day 1]/days of storage)) after storage for 7 days [27]. The characterization of the crumb structure was performed on two bread slices taken from the center of different loaves. The gas cell number of the breadcrumbs was evaluated 24 h after production using image analysis technology [47]. Images of slices of breads (control, SB-SL SWB EPS NEG and SB-SL SWB EPS POS) were scanned full-scale at 300 dots per inch using an image scanner (Amersham Pharmacia Biotech, Uppsala, Sweden). A threshold method was used for differentiating gas cells [52], and the images were analyzed in grey scale (0–255) using the UTHSCSA ImageTool program (Version 2.0, University of Texas Health Science Centre, San Antonio, Texas, available by anonymous FTP from maxrad6.uthscsa.edu). Analysis was carried out on two sub-images with a resolution of 500 × 500 pixels (field of view) selected from within the bread slice. 

### 2.8. Breads Nutritional Characterization

The in vitro starch hydrolysis index (HI) was determined on each type of bread by an enzyme-assisted procedure that mimicked the in vivo digestion [53]. For this analysis, aliquots of breads containing 1 g of starch were subjected to enzymatic digestion. The released glucose content was determined in each sample using a glucose oxidase kit (Megazyme International, Bray, Co., Wicklow, Ireland). Data are expressed as the % of potentially available starch hydrolyzed after 180 min. The predicted glycemic index (pGI) value was then calculated using the equation: pGI = 0.549 × HI + 39.71 [54] with white wheat bread as a reference (HI = 100). 

Total (TDF) and insoluble (IDF) dietary fiber were determined by method AOAC 2011.25 [55]. Soluble (SDF) dietary fiber was calculated as a difference between TDF and IDF according to Tobaruela et al. [55].

### 2.9. Volatile Organic Compounds Profile of Breads

Evaluation of volatile organic compounds (VOCs) was carried out on a Clarus 680 gas chromatography (Perkin Elmer, Beaconsfield UK) equipped with a Rtx-Wax column (30 m × 0.25 mm i.d., 0.25 μm film thickness) (Restek Superchrom, Milano, Italy) coupled to a single-quadrupole mass spectrometer Clarus SQ8MS (Perkin Elmer). The SPME-GC–MS (solid phase micro-extraction gas chromatography–mass spectrometry) protocol and the identification of volatile compounds were performed according to previous reports, with minor modifications [35,56,57]. An amount of 0.750 g of crushed bread (crumb and crust) samples were placed into 20 mL glass vials and added with 10 μL of 4-methyl-2-pentanol (final concentration of 33 mg/L), as the internal standard. A PAL COMBI-xt autosampler (CTC CombiPAL, CTC Analytics AG, Zwingen, Switzerland) was used to standardize the extraction procedure. The samples were then equilibrated for 10 min at 60 °C. The SPME divinylbenzene/carboxen/polydimethylsiloxane (DVB/CARB/PDMS) fiber (Supelco, Bellefonte, PA, USA) was exposed to the sample headspace for 50 min and finally the fiber was inserted into the injection port of the GC at 230 °C to be thermally desorbed and to separate the head space volatile organic compounds. The temperature program was: 35 °C for 8 min, then programmed at 4 °C/min to 60 °C, at 6 °C/min to 160 °C, and finally at 20 °C/min to 200 °C, which was maintained for 15 min. Injections were carried out in splitless mode, and helium (1 mL/min) was used as the carrier gas. The single-quadrupole MS was used to detect the different compounds. The source and transfer line temperatures were 250 and 230 °C, respectively. The MS detector system operated in scan mode with a mass-to-charge ratio interval 35 to 300 Da [57]. Each chromatogram was analyzed for peak identification by comparing (i) retention times with those of pure compounds for HPLC (Sigma-Aldrich, St. Louis, MO, USA) and (ii) experimental mass spectra with those of the National Institute of Standards and Technology database (NIST/EPA/NIH Mass Spectral Library with Search Program, data version NIST 05, software version 2.0d). A peak area threshold of >1,000,000 and a match criterion of >85% were used for VOC identification followed by manual visual inspection of the fragment patterns when required. Quantitative data for the compounds identified were obtained by the interpolation of the relative areas versus the internal standard area.

### 2.10. Bread Sensory Analysis

Sensory analysis of experimental breads after 2–6 h of cooling was carried out by 10 trained panelists (5 males and 5 females, mean age: 30 years, range: 18–54 years) [47]. After a roundtable discussion about the sensory attributes, 15 were selected as the most frequently recognized by all the assessors, which were included in a panel score sheet for the quantitative evaluation using a scale from 0 to 10, with 10 the highest score. Visual and tactual perception (color of crust and crumb, elasticity, consistency, friability), taste (acidic taste, sweetness, salty, legume flavor, bitter flavor), smell perception (acidic odor, caramel-like odor), chewing (chewiness, wetness), and overall aroma were chosen as attributes to characterize the breads. A quarter of each piece of bread sample (including crust and crumb) (1.5 cm thick) were served in random order on a plastic plate encoded with an alpha-numeric code and evaluated by all panelists. Final scores for each attribute were calculated as the means of the data collected during the evaluation.

### 2.11. Statistical Analysis

Experimental data were subjected to analysis of variance (ANOVA); pair-comparison of treatment means was achieved using Tukey’s procedure at *p* < 0.05, using the statistical software Statistica 7.0 for Windows. For each bread, the measured physicochemical (pH, TTA, VOCs)**,** technological, nutritional and sensory characteristic data were used as variables for the Principal Component Analysis (PCA).

## 3. Results and Discussion

### 3.1. pH and Viscosity in Preliminary Liquid Sourdough Formulations

Preliminarily, different doughs were produced using native barley (B) or sprouted barley (SB) flour or blend of SB with native (L) or sprouted lentil (SL) flour at different ratio (40:60 or 30:70% *w*/*w*) during sucrose-supplemented (5% on dough weight, corresponding to 16% flour weight, f.w.) or without sucrose addition fermentation. The germination protocols used in this study to obtain sprouted flours from barley and lentil grains were set-up in previous studies [33,35,45]. The doughs were singly inoculated with the dextran-producer *W. confusa* SLA4, *W*. *paramesenteroides* SLA5, *L. pseudomesenteroides* DSM 20193 and *W. confusa* DSM 20194 strains [26,27,35,36,44], and by *L. plantarum* DPPMAB24W, a dextran not producer strain [44], and fermented for 24 h at 20 or 25 °C. These trials were performed to select the best strains and parameters leading the significant increase in the viscosity in the fermented doughs. The pH and viscosity were analyzed to show the influence of acid and dextran production on the rheological properties of the sourdoughs. Viscosity formation during fermentation indicates the presence of large molecules with water binding properties and correlates with in situ EPS synthesis [18,19]. After 24 h of fermentation, the values of pH became lower than ca. 4.3 in almost all the sourdoughs (Table S2). All the sourdoughs exhibited a shear thinning behavior. The viscosity of all sourdoughs increased after 24 h of fermentation compared to the *L. plantarum* DPPMAB24W fermented doughs. Both the *Weissella* spp. and *L. pseudomesenteroides* sucrose-supplemented sourdoughs exhibited significantly higher viscosities than their EPS-negative counterparts. When fermented with selected strains, sourdoughs containing only native or sprouted barley flour showed low viscosity increase, presumably due to low dextran production, and, therefore, were not further considered. This effect might be due to the high content of maltose or other sugar acceptors which might have favored the formation of low-molecular mass oligosaccharides from sucrose rather than dextran, as typically occurs in cereal substrate, leading to a low viscosity formation [18,19,58,59] and/or to the low pH reached in those sourdoughs (pH average ca. 3.78), also affecting viscosity. 

The viscosity of the blends of sprouted barley and native (SB-L) or sprouted lentil (SB-SL) was ca.0.3 Pa*s before fermentation, with similar values between EPS POS and EPS NEG doughs, while ranged from 0.15 to 0.96 Pa *s in EPS NEG and from 0.17 to 3.33 Pa*s in EPS POS sourdoughs after fermentation, with the highest viscosity recorded for SB-SL EPS POS indicating the thickening ability of EPS [44] and the successful outcome of the mixture cereal/legume. Blend of SB-L and SB-SL flours at 30:70% *w*/*w* ratio and fermented by *L. pseudomesenteroides* DSM 20193 at 20 °C per 24 h, DY333, showed the highest viscosity (2.90 and 3.30 Pa/s for SB-L and SB-SL sourdoughs, respectively) (Table S2). Hence, SB-L and SB-SL blends at 30:70% *w*/*w* ratio fermented by *L. pseudomesenteroides* DSM 20193 at 20 °C per 24 h, DY333, were selected and further characterized. 

### 3.2. Microbial Growth and Acidification in the Liquid Sourdough Formulations

Microbial profiles of selected sourdoughs changed after the fermentation, but no significant differences were observed between EPS POS and EPS NEG sourdoughs. The initial cell count of lactic acid bacteria and total mesophilic aerobic bacteria in all sourdoughs was approximately 6.7 log cfu/g (Table 1).

In agreement with earlier studies [35], LAB cell density increased ca 2.5 log cycle after 24 h of fermentation, independently of sucrose presence, indicating that SB-L and SB-SL are good substrates for the growth of the strain used. The mesophilic bacteria count in SB-L sourdough was slightly (*p* < 0.05) lower than SB-SL sourdoughs after 24 h. Before fermentation, *Enterobacteriaceae* were higher than 5 log cfu/g in SB-SL sourdoughs, while were ca. 2 log cfu/g in all SB-L sourdoughs. In all cases, any significant difference (*p* <0.05) among EPS POS and EPS NEG sourdough was observed. After 24 h, *Enterobacteriaceae* grew up to four log cycles. The highest increase was observed in SB-L sourdoughs. The initial cell density of yeasts in all sourdoughs was lower than 3 log cfu/g. In the SB-SL sourdoughs, the yeast cell density was 1 log cfu/g higher than SB-L doughs. After fermentation, yeasts were not detected. Before fermentation, the pH of all the doughs was around 6.15; it decreased by ca. 1.7 units after 24 h of fermentation, reaching values in the range from 4.4 to 4.6 (Table 1). The fermentation drove to an increase in the TTA in all the sourdough types. TTA values varied from 11.6 to 14.2 mL, with the highest value for SB-SL EPS POS. Fermentation caused an increase in the concentration of both lactic and acetic acids (Table 1). Lactic acid concentration ranged from 18 to 22.0 mmol/kg and was slightly higher in both SB-SL sourdoughs compared to SB-L doughs. The highest value for acetic acid was found in SB-SL EPS POS. The concentration of lactic and acetic acid in sourdoughs plays a central role in the flavor and taste of sourdough bread. Sucrose addition, through the liberation of fructose, facilitated the production of acetic acid while lactic acid concentration decreased, which was also represented by the fermentation quotient (FQ). It has been described that *Leuconostoc* spp. can reduce the released fructose to mannitol, taking part at the acetic acid formation [60]. FQ was calculated as the molar ratio between lactic acid and acetic acid, and it ranged between 2.59 in SB-SL EPS POS and 4.11 in SB-L EPS NEG, indicating that the addition of sucrose lead to a lower FQ. 

### 3.3. Sugars and Dextran Content in the Liquid Sourdough Formulations

**Figure 1:**
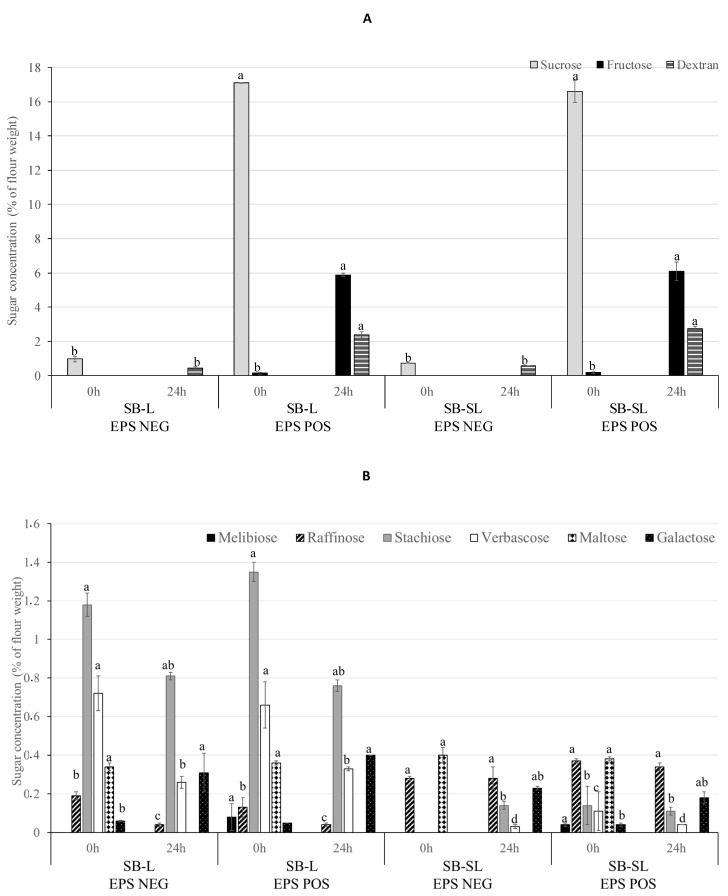
Sugars and dextran concentration (% of flour weight) in control and sucrose-supplemented (16% of flour weight) doughs (DY 333) before and after 24 h of fermentation at 20 °C started by *Leuconostoc pseudomesenteroides* DSM 20193 (initial cell density of ca. 7 log cfu/g). Panel (**A**) shows sucrose, fructose and dextran concentration; panel (**B**) shows melibiose, raffinose, stachyose, verbascose, maltose and galactose concentration. SB-L EPS NEG, sprouted barley-native lentil dough (30:70 ratio); SB-L EPS POS, sucrose-supplemented sprouted barley-native lentil dough (30:70 ratio); SB-SL EPS NEG, sprouted barley–sprouted lentil dough (30:70 ratio); SB-SL EPS POS, sucrose-supplemented sprouted barley–sprouted lentil dough (30:70 ratio). For each sugar, bars with different superscript letters differ significantly (*p* < 0.05).

The amount of extractable free sugars in all sourdoughs before and after 24 h of fermentation is shown in Figure 1.

SB-L and SB-SL doughs naturally contained ca. 0.97–0.74% f.w. of sucrose and 0.34–0.4% f.w. of maltose, respectively, (Figure 1A,B) and the supplementation of sucrose (5% on dough weight, corresponding to 16% f.w.) was necessary to obtain enough EPS yield by *L. pseudomesenteroides* DSM 20193 [15]. After fermentation, sucrose was totally used by *L. pseudomesenteroides* DSM 20193. The low amount of dextran formed in EPS NEG doughs (0.43–0.58% flour basis) may be formed from the sucrose existent in the native flour. *L. pseudomesenteroides* DSM 20193 in SB-L and SB-SL sucrose enriched doughs produced up to 2.7% f.w. of dextran, which is less than the theoretical. Based on sucrose addition, up to 8% f.w. of dextran could be formed. Previously, dextran-forming *L. pseudomesenteroides* DSM 20193 produced 3.6% of dextran on a wet weight base in faba bean sourdough upon the addition of 25% f.w. sucrose [44,46]. The yield of EPS is resultant of sucrose content, the presence of acceptors molecules, such as maltose, and the starter strain and growth conditions, amongst others [61]. Based on the sugar analysis, glucose released from added sucrose was utilized by *L. pseudomesenteroides* DSM 20193 only partly for dextran production. The glucansucrase acted on sucrose-synthesizing glucan and liberating fructose, as confirmed by the residual fructose in all sucrose-containing doughs, in the range from 5.89% to 6.09% [60]. 

Together with the increase in viscosity, the capacity of *L. pseudomesenteroides* DSM 20193 to reduce different Raffinose Family Oligosaccharides (RFO), such as raffinose, stachyose and verbascose, during fermentation of the blend of SB-L and SB-SL flours was investigated, since they are one of the main limits to the use of legumes in animal and human nutrition [62]. RFOs were detected at different extends in all doughs before fermentation (Figure 1B), without significant difference among EPS NEG and EPS POS samples. Raffinose, as expected, was not affected by germination and was contained in both the blends [51]. Raffinose cannot be broken down by human digestive enzymes but can be utilized by anaerobic bacteria in the large intestine, thus, causing the production of flatus gases and gastrointestinal discomfort. Nevertheless, stachyose and verbascose concentration was very low in SB-SL compared to SB-L (Figure 1B), which can be attributed to the more intensive effect of different endogenous α-galactosidases during sprouting. RFOs can be enzymatically hydrolyzed by LAB during fermentation [63,64], thus, increasing product digestibility and reducing digestive discomfort [65]. Fermentation with *L. pseudomesenteroides* DSM 20193 decreased the RFOs content in both SB-L and SB-SL sourdoughs and particularly in the SB-L flour sourdough. Fermentation significantly increased (*p* < 0.05) galactose, a degradation product of RFO by α galactosidase, in all doughs.

### 3.4. Breads Characterization

**Figure 2:**
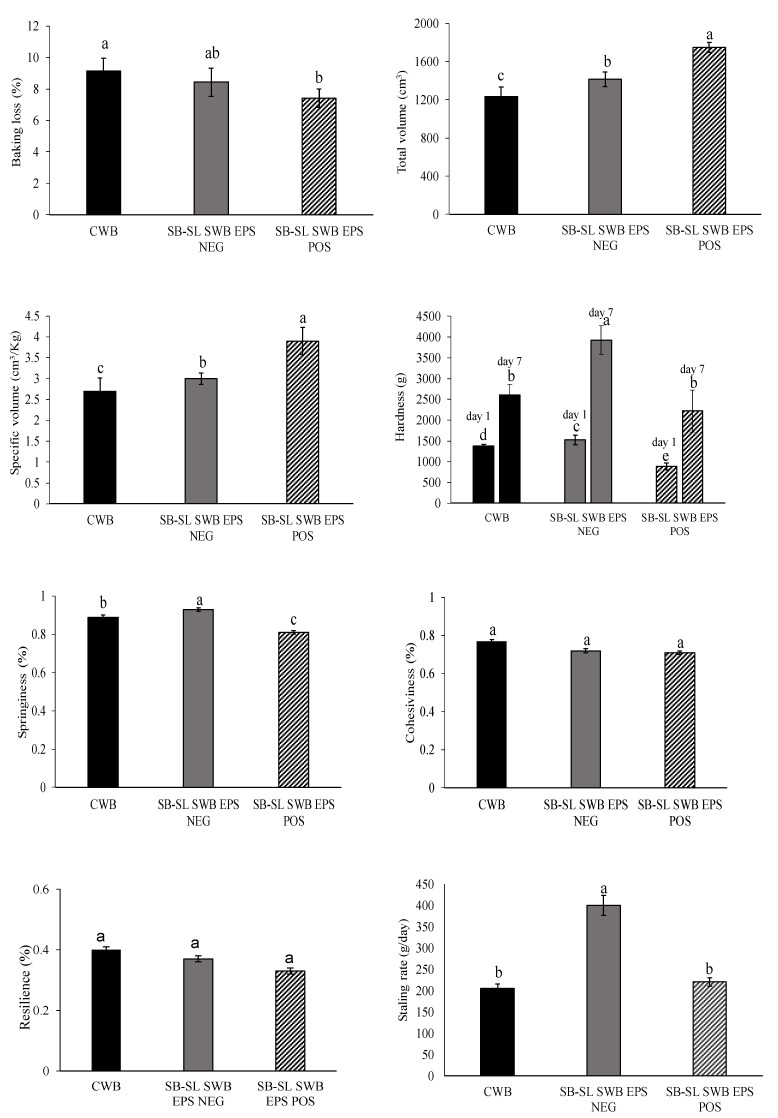
Technological characteristics of control and sourdough breads (DY 162). CWB, control wheat bread started with baker’s yeast; SB-SL SWB EPS NEG, bread containing wheat flour added with 30% (*w*/*w*) sprouted barley–sprouted lentil (30:70% *w*/*w*) sourdough fermented by *Leuconostoc pseudomesenteroides* DSM 20193; SB-SL SWB EPS POS, bread containing wheat flour added to 30% (*w*/*w*) dextran-containing sprouted barley–sprouted lentil (30:70% *w*/*w*) sourdough fermented by *L. pseudomesenteroides* DSM 20193. The initial cell density of the strain was ca. 7 log cfu/g. The sourdough was fermented at 20 °C for 24 h. For each bread, bars with different superscript letters differ significantly (*p* < 0.05).

Considering the higher in situ production of dextran and the balanced acid production, SB-SL sourdoughs were selected for subsequent baking trials. The content of dextran synthesized in situ in SB-SL sourdough by *L. pseudomesenteroides* DSM 20193 was 2.73% of flour basis and consequently the correspondent final breads contained 0.36% flour basis of dextran, which is in the range (0.1–2%) of the number of commercial hydrocolloids such as CMCHPMC, GG and κ-CAR applied in baking [66]. Fermentation and bread making were carried out applying the process parameters typical for type I sourdough fermentation [47]. The addition of dextran-containing sourdoughs into bread significantly affected the technological features compared to the control bread (Figure 2). 

Compared to CWB, the substitution of wheat with dextran-enriched or EPS NEG SB-SL fermented doughs, resulted in a significant (*p* < 0.05) increase in the bread volume accompanied by a decrease in baking loss (37% of baking loss in SB-SL SWB EPS POS bread), leading to a superior quality of the wheat bread. The use of dextran-enriched sourdough increases the specific volumes compared to breads fortified with the corresponding EPS NEG counterpart. The presence of dextran significantly decreased the hardness of the composite bread. After 1 day, the hardness of bread crumb was significantly (*p* < 0.05) lower in SB-SL SWB EPS POS (892 ± 79 g) when compared to CWB and their respective negative EPS NEG counterparts. Furthermore, the hardness of SB-SL SWB EPS NEG was higher (*p* < 0.05) than CWB. After 7 days of storage, the hardness of dextran-enriched SWB (2218 g) was lower but not significantly different (*p* >0.05) compared to CWB (2610 g), while strongly higher was the hardness of SB-SLS WB EPS NEG (3927 g) (Figure 2). It is known that the use of grains different from wheat or legumes markedly affect the properties of baked goods [6], usually leading to a weak dough structure and baking quality, decreased bread volume and elasticity of the crumb and to an increased hardness of the loaves [38,67]. Springiness is how well a product physically springs back after it has been deformed. A lower value of springiness was observed for SB-SL SWB compared to CWB. Cohesiveness is how well the bread withstands a second deformation relative to its resistance under the first deformation. Resilience is how well a product fights to regain its original height. Moreover, for these two parameters, SB-SLSWB EPS POS showed the lowest value (Figure 2). The staling rate, as determined by TPA analysis, was affected (*p* < 0.05) by the addition of sourdoughs and followed the order SB-SL SWB EPS NEG > SB-SL SWB EPS POS > CWB. Bread staling involves changes in both crumb and crust and is a complex and physicochemical irreversible phenomenon involving starch amylopectin recrystallization and water redistribution [68]. The anti-staling effect of hydrocolloids, such as dextran, could be related to dextran polymers competing for water, so that less water molecules are available for the development of amylopectin crystallites [69]. Similar findings were reported in recent works studying the effect of the addition of dextran to faba bean–wheat, pearl millet–wheat and lentil-or sprouted lentil–wheat composite bread [26,27,35]. Image analysis technology was performed on crumb grain of bread slices after 24 h of storage to provide a more detailed view of the bread texture, Digital images were pre-processed to detect crumb cell total area by a binary conversion (black/white pixels). The gas cell-total area (corresponding to the black pixel ratio) of the breads containing sourdough were significantly (*p* < 0.05) higher than CWB. Crumb cell detection of bread slice portions showed that no significant difference in the mean area of gas cells could be observed between SB-SL SWB EPS NEG and SB-SL SWB EPS POS, having values of 56.4 ± 0.11 and 59.1 ± 0.08% pixels, respectively. The pH of the CWB bread crumb after baking was 5.92 ± 0.111, higher (*p* < 0.05) than both the sourdough breadcrumbs (4.68 ± 0.202 and 4.72 ± 0.137 for EPS POS and EPS NEG, respectively). All doughs containing sourdoughs presented TTA values significantly higher (*p* < 0.05) (13.6 ± 0.303 and 15.2 ± 0.093 mL NaOH 0.1N for SB-SL SWB EPS NEG and SB-SL SWB EPS POS breads, respectively) than the control bread (5.20 ± 0.404). 

### 3.5. Dietary Fiber and Starch Hydrolysis Index

**Figure 3:**
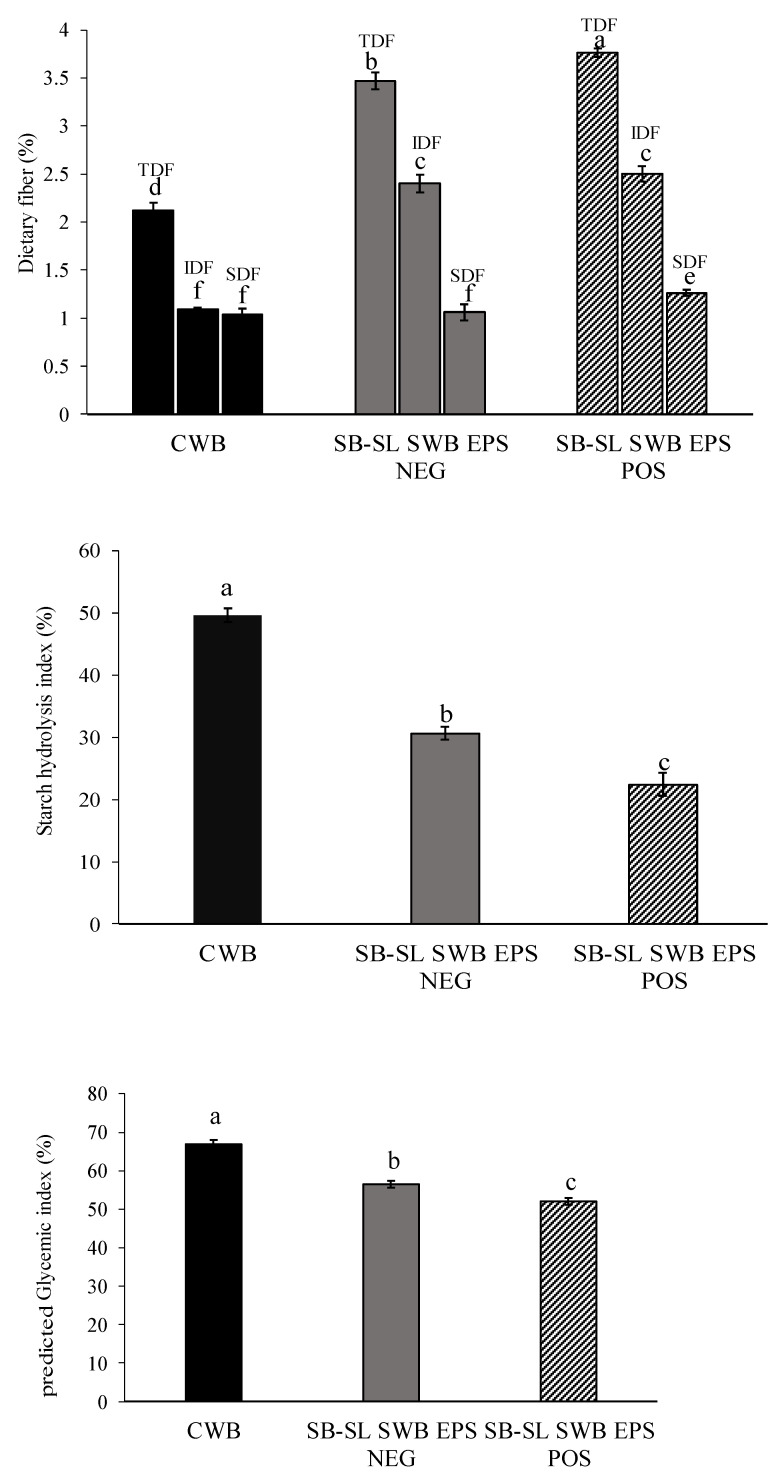
Nutritional characteristics of control and sourdough breads (DY 162). CWB, control wheat bread started with baker’s yeast; SB-SL SWB EPS NEG, bread containing wheat flour added with 30% (*w*/*w*) sprouted barley–sprouted lentil (30:70% *w*/*w*) sourdough fermented by *Leuconostoc pseudomesenteroides* DSM 20193; SB-SL SWB EPS POS, bread containing wheat flour added to 30% (*w*/*w*) dextran-containing sprouted barley–sprouted lentil (30:70% *w*/*w*) sourdough fermented by *L. pseudomesenteroides* DSM 20193. fibers. The initial cell density of the strain was ca. 7 log cfu/g. The sourdough was fermented at 20 °C for 24 h. TDF, total dietary fibers; IDF, insoluble dietary fibers; SDF, soluble dietary. For each bread, bars with different superscript letters differ significantly (*p* < 0.05).

Regarding the nutritional value, the incorporation of 30% EPS NEG and dextran-containing SB-SL sourdoughs led to a significant increase in total dietary fibers (TDF) compared to CWB, which may be beneficial for consumers’ health (Figure 3). 

The dextran-containing SB-SL bread had the highest content of SDF compared to the corresponding EPS NEG and CWB breads which can be due to the dextran and oligosaccharides produced. According to EC Regulation No 1924/2006 [48] on nutrition and health claims on food products, the composite grain breads can be labelled as “source of fiber”, since containing at least 3 g of fiber/100 g of bread. 

The predicted glycemic index (pGI) of breads containing sourdough from sprouted grains was lower than the reference bread. It is well known that sprouting can increase the release of reducing sugars and, consequently, HI and pGI thanks to starch hydrolysis by alpha-amylase and beta-amylase enzymes [70,71]. However, the germination conditions used in this work (16 °C and 3 days), such as those reported previously [32], may be useful to produce sprouted flours with a low glycemic index. Moreover, sourdough fermentation, per se, decreased the HI through the synthesis of organic acids [72]. The pGI value of dextran-containing SB-SL SWB bread (52%) was lower (*p* < 0.05) than the corresponding EPS NEG (56.5%) and the CWB (66.9%) (Figure 3). This could be attributed to the effects of β-glucans soluble fibers from barley on reducing GI [39] which the content here could be 0.05–0.27% of dough weight (based on 2–10% beta-glucan content of barley) [73] and high concentration of fibers of the legume flours [47]. β-glucans are non-starch polysaccharides composed of glucose molecules in long linear polymers with mixed β-(1→4) and β-(1→3) links (from 30% to 70%). Their *MW* ranges from 50 to 2000 kDa. The mixed linkages are important for their solubility and viscosity properties, and their viscosity is a function of the content of dissolved β-glucans, and of their *MW* [74], and further depends on differences on raw materials and processing [39]. Although the β-glucan content, solubility, viscosity, and *MW* were not considered in this study, some considerations should be made. The fermentation and bread-making process partially affects β-glucan level and *MW* [75,76]. A significant degradation of β-glucan occurring during fermentation may reduce the nutritional functionality of the residual β-glucan since it is supposed to be dependent on *MW*. High *MW* and insoluble β-glucan are considered positive for various physiological functions [38,40,74], comprising the reduction of glycemic response by increasing the viscosity of food. Foods with increased viscosity have been demonstrated to increase gastric transition time and slow absorption [38,77]. 

### 3.6. Volatile Organic Compounds

HS-SPME-GC–MS analysis was applied to characterize bread VOCs. Sixty-nine VOCs were found. Significantly different VOCs were grouped into ten different chemical classes (Table 2). 

The addition of SB-SL sourdough to the bread caused significant (*p* < 0.05) changes in bread VOCs compared to CWB with positive repercussions on the global aroma profile of bread, influencing consumer acceptance [78]. As previously reported, composite sourdough wheat breads exhibited more complex aroma volatile profiles compared to bakers’ yeast breads (Table 2) [79,80]. SB-SL SWB breads presented a higher concentration of alcohols and organic acids compared to CWB. Among the seven alcohol compounds, ethanol, 1-hexanol alcohol, and benzylalcohol were the most abundant. Acetic acid and hexanoic acid were the most representative carboxylic acids, especially in SB-SL SWB breads. Furfural was the most abundant aldehyde, particularly in SB-SL SWB EPS POS bread. 2-methylpropanal, 2-methylbutanal, 3-methylbutanal, benzaldehyde and 2-nonenal were the most abundant among the 11 aldehydes found in the SB-SL SWB breads. In general, the addition of sourdoughs did not affect the ethylacetate ester content; on the contrary, the concentration of acetoin (ketone) was affected and it appeared more abundant in CWB. A total of 12 heterocyclic compounds significantly differed among breads, 2-pentyl-furan and 2-methylpyrazine being the most representative. Moreover, in SB-SL SWB was observed a higher concentration of maltol. Dextran-containing SB-SL bread and its negative counterpart showed similar VOCs content. Therefore, these two breads should have, theoretically, received comparable scores for legume and bitterness flavor intensity. However, the SB-SL SWB EPS POS bread was perceived as more sweet, likely for the effect of the residual reducing sugars which contribute to the sweet taste and promote during baking the complex of Maillard reactions and the caramelization [28]. Wang et al. [28] reported a flavor-masking effect for dextran-enriched sorghum sourdough bread (0.56% bread weight) which showed decreased perception of bitterness, sourness, and aftertaste, compared to EPS NEG sorghum sourdough bread. The concentration of odorants in the final product can be driven by the ingredients and baking process [80] and sprouting and sourdough fermentation can significantly modify flavor and texture of raw materials [81]. Moreover, other volatile compounds with different origins are generated during baking [82]. 

### 3.7. Sensory Profile

**Figure 4:**
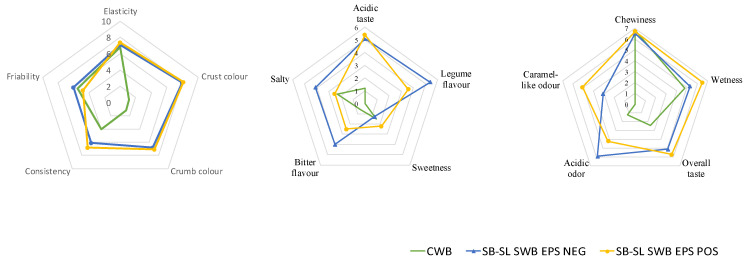
Spider web chart of the sensory analysis data for control and sourdough breads (DY 162). CWB, control wheat bread started with baker’s yeast; SB-SL SWB EPS NEG, bread containing wheat flour added with 30% (*w*/*w*) sprouted barley–sprouted lentil (30:70% *w*/*w*) sourdough fermented by *Leuconostoc pseudomesenteroides* DSM 20193; SB-SL SWB EPS POS, bread containing wheat flour added to 30% (*w*/*w*) dextran-containing sprouted barley–sprouted lentil (30:70% *w*/*w*) sourdough fermented by *L. pseudomesenteroides* DSM 20193. The initial cell density of the strain was ca. 7 log cfu/g. The sourdough was fermented at 20 °C for 24 h.

Overall, sourdough breads achieved higher scores for several attributes such as elasticity, color, acidic taste and acidic odor compared to the CWB (Figure 4). 

Sourdough fermentation had an impact on flavor perception of final breads, which resulted in a bitter taste and legume flavor probably originating by liberation of small molecular weight polyphenol, bitter peptides and amino acids and the intense acidification due to endogenous and/or microbial enzymatic activities, and that could not be appreciated by the consumers [28,83]. Dextran-containing SB-SL bread was characterized by consistency, sweetness and caramel-like odor with significantly less perceived legume flavor and bitterness, while EPS NEG was judged as the most friable by the panelists, highlighting the fundamental role of dextran in improving bread texture and masking of unpleasant notes in composite bread [28]. As already reported, fiber-rich baked goods could help to reach the recommended dietary fiber intake, but they typically have a lower sensorial quality compared to baked goods produced from more refined ingredients [84]. In spite of this, composite SB-SL sourdough breads were more appreciated than CWB, as in previous studies [33,47].

### 3.8. Correlations between VOCs, Technological, Nutritional and Sensory Features of Breads

**Figure 5:**
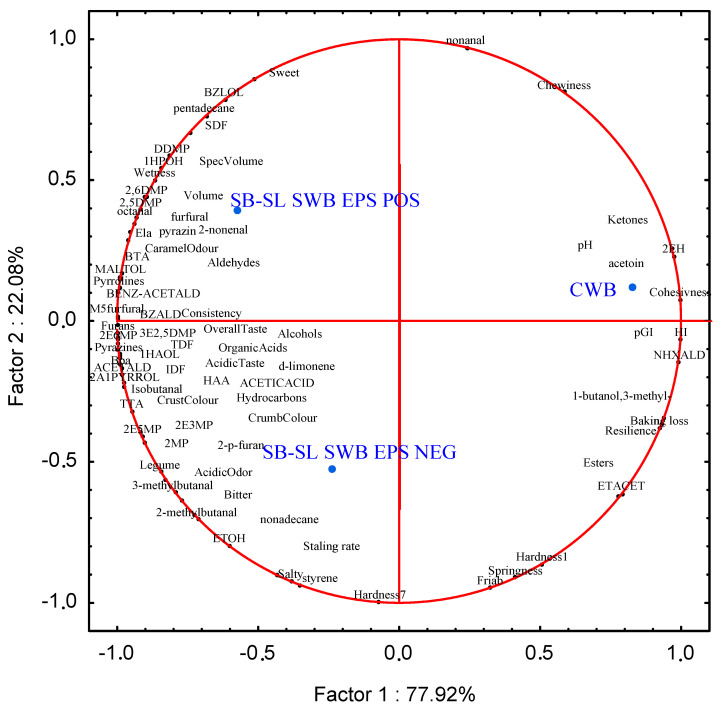
Correlations between physicochemical (pH, TTA, VOCs)**,** technological, nutritional and sensory features of control and sourdough breads (DY 162). CWB, control wheat bread started with baker’s yeast; SB-SL SWB EPS NEG, bread containing wheat flour added with 30% (*w*/*w*) sprouted barley-sprouted lentil (30:70% *w*/*w*) sourdough fermented by *Leuconostoc pseudomesenteroides* DSM 20193; SB-SL SWB EPS POS, bread containing wheat flour added to 30% (*w/w*) dextran-containing sprouted barley–sprouted lentil (30:70% *w*/*w*) sourdough fermented by *L. pseudomesenteroides* DSM 20193. The initial cell density of the strain was ca. 7 log cfu/g. The sourdough was fermented at 20 °C for 24 h. pH, pH bread crumb; TTA, TTA bread crumb; ETOH, ethanol; isopentanol, 1-butanol,3-methyl-; 1HAOL, 1-hexanol; 1HPOH, 1-heptanol; 2EH, 2-ethylhexanol; BZLOL, benzylalcohol; BTA, butanoic acid; HAA, hexanoic acid; NHXALD, hexanal; BZALD, benzaldehyde; BENZ-ACETALD, benzeneacetaldehyde; ETACET, ethylacetate; DDMP, 4 h-pyran-4-one,2,3-dihydro-3,5; 2-p-furan, 2-pentyl-furan; 2MP, 2-methylpyrazine; pyrazin, pyrazinamide; 2,5DMP, 2,5-dimethylpyrazine; 2,6DMP, 2,6-dimethylpyrazine; 2E6MP, 2-ethyl-6-methylpyrazine; 2E5MP, 2-ethyl-5-methylpyrazine; 2E3MP, 2-ethyl-3-methylpyrazine; 3E2,5DMP, 3-ethyl-2,5-dimethylpyrazine; 2A1PYRROL, 2-acetyl-1-pyrroline; MALTOL, 3-hydroxy-2-methyl-4-pyrone; Bpa, black pixel area; HI, hydrolysis index; pGI, predicted glycemic index; Ela, elasticity; Friab, friability; Legume, legume flavor; Sweet, sweetness; Bitter, bitter flavor.

Relationship among measured chemical, technological, nutritional and sensory parameters from CWB and composite SWB breads, which could affect consumer acceptance were elaborated through Principal Component Analysis (PCA) (Figure 5). 

The two PCs explained ca. 100% of the total variance of the data. Composite sourdough breads showed peculiar profiles and fell into different zones of the plane. Factor 1 clearly separated SB-SL SWB EPS NEG from dextran-containing bread. Factor 2 differentiated SWB and control breads. The chewiness and cohesiveness together with the highest values of some VOCs (e.g., total ketones, acetoin, 2-ethylhexanol) and nutritional (hydrolysis index and predicted GI) data, mainly characterized CWB. The dextran-containing SB-SL SWB not grouped together the EPS NEG because of the high volume, sweetness, wetness of the crumb, SDF, low hardness and springiness. The SB-SL SWB EPS NEG bread separated from the EPS POS for the higher hardness after 1 and 7 days, staling rate, legume and bitter flavor. Overall, the incorporation of 30% dextran-enriched SB-SL sourdough confirmed that the synergistic use of the sprouting process and LAB fermentation improves the nutritional and functional quality of cereal and legume grains with a positive effect on the undesirable beany flavor [33,35] compared to the EPS NEG counterpart.

## 4. Conclusions

In conclusion, the bioprocessing procedure developed successfully improved the quality of composite wheat bread, creating a good nutritional and sensory quality. The modulation of the fermentation parameters (native or sprouted cereal and legume flour type, DY, temperature) can stimulate metabolic activities of selected LAB strains, making them suitable for the production of sourdoughs enriched in EPS, which can be applied in the bread-making process as a “clean label” strategy. 

The amount of dextran produced by *L. pseudomesenteroides* DSM20193 in a blend of sprouted barley and sprouted lentil flours effectively counteracted the quality deficiencies induced by wheat flour substitution in the composite sourdough bread. Dextran-enriched sprouted barley–sprouted lentil sourdough, resulting in the best overall system, could be used at a high level (30% of the dough weight) in wheat bread baking, resulting in bread with an enhanced nutritional quality (low HI and pGI), functionality (high soluble and total fibers content) and appreciable sensory attributes. 

## Figures and Tables

**Table 1:** Microbial growth and acidification ^1^ of control (EPS NEG) and sucrose-supplemented (16% of flour weight) doughs (DY 333) before and after 24 h of fermentation at 20 °C started by *Leuconostoc pseudomesenteroides* DSM 20193 (initial cell density of ca. 7 log cfu/g). The table shows microbial cell density (log cfu/g) of LAB, total mesophilic bacteria, *Enterobacteriaceae* and yeasts, pH, TTA (mL), lactic acid and acetic acid concentrations (mmol/Kg dough) and their ratios (FQ). SB-L EPS NEG, sprouted barley-raw lentil dough (30:70 ratio); SB-L EPS POS, sucrose-supplemented sprouted barley-raw lentil dough (30:70 ratio); SB-SL EPS NEG, sprouted barley–sprouted lentil dough (30:70 ratio); SB-SL EPS POS, sucrose-supplemented sprouted barley–sprouted lentil dough (30:70 ratio).

Sample Code	Lactic Acid Bacteria	ΔLog ^a^	Total Mesophilic Bacteria	*Enterobacteriaceae*	Yeasts	pH	TTA	Lactic Acid	Acetic Acid	FQ ^b^
	T0 h									
**SB-L EPS NEG**	6.81 ± 0.107 a		6.62 ± 0.117 b	1.77 ± 0.097 d	<1 c	6.27 ± 0.113 a	4.18 ± 0.093 d	n.d. ^c^	n.d.	-
**SB-L EPS POS**	6.84 ± 0.058 a		6.53 ± 0.143 b	1.53 ± 0.176 d	1.14 ± 0.176 b	6.22 ± 0.044 a	4.57 ± 0.117 d	n.d.	n.d.	-
**SB-SL EPS NEG**	6.78 ± 0.192 a		6.74 ± 0.117 b	5.44 ± 0.097 b	2.35 ± 0.087 a	6.07 ± 0.095 a	5.4 ± 0.135 c	n.d.	n.d.	-
**SB-SL EPS POS**	6.71 ± 0.144 a		6.72 ± 0.033 b	5.53 ± 0.176 b	2.29 ± 0.014 a	6.15 ± 0.085 a	5.6 ± 0.110 c	n.d.	n.d.	-
	T24 h									
**SB-L EPS NEG**	9.51 ± 0.044 a	2.71 ± 0.035 a	8.87 ± 0.111 a	4.81 ± 0.063 c	<1 c	4.62 ± 0.115 b	11.6 ± 1.241 b	18.00 ± 0.10 c	4.38 ± 0.10 c	4.11± 0.18
**SB-L EPS POS**	9.35 ± 0.148 a	2.51 ± 0.055 a	9.03 ± 0.201 a	5.01 ± 0.212 c	<1 c	4.53± 0.271 b	12.4 ± 0.115 b	17.20 ± 0.72 c	5.45 ± 0.22 b	3.16 ± 0.13
**SB-SL EPS NEG**	9.43 ± 0.152 a	2.65 ± 0.082 a	9.14 ± 0.257 a	5.81 ± 0.091 a	<1 c	4.44 ± 0.095 bc	13.6 ± 0.241 ab	22.00 ± 0.10 a	5.40 ± 0.10 b	4.07 ± 0.18
**SB-SL EPS POS**	9.55 ± 0.074 a	2.84 ± 0.112 a	9.23 ± 0.151 a	6.01 ± 0.151 a	<1 c	4.42 ± 0.045 bc	14.2 ± 0.115 a	20.20 ± 0.10 b	7.80 ± 0.22 a	2.59 ± 0.13

^1^ Data are mean values ± standard deviation. a–d, means within a column with different letters are significantly different (*p* < 0.05). ^a^ The increase in LAB cell density after 24 h of fermentation. ^b^ FQ, fermentation quotient. ^c^ n.d., not detected.

**Table 2:** Quantification of total volatile organic compounds (VOCs) divided by chemical classes in control and sourdough breads (DY 162). Normalized data with the internal standard are reported, calculated as ratio peak area/total peak area percent. CWB, control wheat bread started with baker’s yeast; SB-SL SWB EPS NEG, bread containing wheat flour added with 30% (*w*/*w*) sprouted barley–sprouted lentil (30:70%) sourdough fermented by *Leuconostoc pseudomesenteroides* DSM 20193; SB-SL SWB EPS POS, bread containing wheat flour added to 30% (*w*/*w*) dextran-containing sprouted barley–sprouted lentil (30:70%, *w*/*w*) sourdough fermented by *L. pseudomesenteroides* DSM 20193. The initial cell density of the strain was ca. 7 log cfu/g. The sourdough was fermented at 20 °C for 24 h.

Compounds	Odor	CWB	SL-SB SWB EPS NEG	SL-SB SWB EPS POS
***Alcohols***				
Ethanol	Alcoholic	2.03 ± 0.096 b	3.65 ± 0.074 a	2.7 ± 0.109 b
3-methylbutanol	Balsamic, alcoholic, malty	0.65 ± 0.014 a	0.52 ± 0.025 ab	0.36 ± 0.004 b
1-hexanol	Green grass, woody, sweet, flowery, mild	0.18 ± 0.014 b	1.00 ± 0.163 a	1.18 ± 0.031 a
1-heptanol	Green	n.d. ^1^	0.01 ± 0.001 b	0.04 ± 0.015 a
2-ethylhexanol	Green, vegetable	0.27 ± 0.026 a	n.d.	n.d.
Benzylalcohol	Pleasant, aromatic	0.34 ± 0.135 b	0.39 ± 0.041 b	0.90 ± 0.016 a
1-nonanol	Citrus	0.01 ± 0.003 b	0.19 ± 0.089 a	0.27 ± 0.005 a
*Total*		3.56 ± 0.13	5.8 ± 0.63	5.45 ± 0.19
***Organic Acids***				
Acetic acid	Sour, acid, pungent	0.05 ± 0.004 b	0.50 ± 0.201 a	0.56 ± 0.013 a
Butanoic acid	Sweaty, rancid	n.d.	0.02 ± 0.001	0.03 ± 0
Hexanoic acid	Sweaty, cheesy, fatty, goat-like	0.04 ± 0.001 b	0.26 ± 0.073 a	0.28 ± 0.012 a
*Total*		0.10 ± 0.02 b	0.78 ± 0.03 a	0.87 ± 0.43 a
***Aldehydes***				
2-methylpropanal	Malty	0.1 ± 0.026 b	0.19 ± 0.003 a	0.19 ± 0.009 a
2-methylbutanal	Almond, malty	0.01 ± 0.001 b	0.24 ± 0.078 a	0.13 ± 0.015 ab
3-methylbutanal	Malty, roasty, cucumber-like	0.01 ± 0.003 b	0.37 ± 0.122 a	0.23 ± 0.01 ab
Hexanal	Green, grassy	0.52 ± 0.017 a	0.39 ± 0.069 b	0.33 ± 0.015 b
Octanal	Citrus, flowery	0.02 ± 0.029 b	0.18 ± 0.161 ab	0.34 ± 0.032 a
Nonanal	Citrus, soapy	0.37 ± 0.036a	0.26 ± 0.061b	0.44 ± 0.016a
Furfural	Almond, soil	0.26 ± 0.020b	1.11 ± 0.430a	1.90 ± 0.039a
Benzaldehyde	Almond, caramel	0.07 ± 0.009b	0.32 ± 0.08a	0.38 ± 0.002a
2-nonenal	Fatty, tallowy, green	0.02 ± 0.006b	0.17 ± 0.076ab	0.28 ± 0.002a
Acetaldehyde	Fruity	0.05 ± 0.003b	0.23 ± 0.074a	0.24 ± 0.003a
Benzeneacetaldehyde	flowery, honey-like	n.d.	0.02 ± 0.008a	0.03 ± 0.002a
*Total*		1.43 ± 0.03b	3.48 ± 0.72ab	4.49 ± 0.10a
***Esters***				
Ethylacetate	Sweet, fruity, pineapple	0.09 ± 0.003ab	0.11 ± 0.05a	0.05 ± 0b
*Total*		0.09 ± 0.00 ab	0.11 ± 0.00 a	0.05 ± 0.05 b
***Ketones***				
Acetoin	Butter, butterscotch, cream, yogurt	0.12 ± 0.005 a	0.06 ± 0.015 b	0.06 ± 0.009 b
*Total*		0.12 ± 0.005 a	0.06 ± 0.015 b	0.06 ± 0.009 b
***Hydrocarbons***				
d-limonene	Citrus	0.04 ± 0.032 b	0.18 ± 0.011 a	0.19 ± 0.031 a
Styrene	Pungent	n.d.	0.06 ± 0.013 a	0.01 ± 0.021 b
Nonadecane	n.f. ^2^	n.d.	0.02 ± 0.014 a	0.01 ± 0.017 a
Pentadecane	n.f.	n.d.	n.d.	0.02 ± 0 ab
4 h-pyran-4-one,2,3-dihydro-3,5	Caramelized	n.d.	0.01 ± 0.014 b	0.05 ± 0.008 a
*Total*		0.04 ± 0.032 b	0.27 ± 0.07 a	0.28± 0.03 a
***Furans***				
2-pentyl- furan	Butter, green bean, floral	0.08 ± 0.024 b	0.32 ± 0.278 ab	0.28 ± 0.063 a
2-furancarboxaldehyde,5-methyl	n.f.	0.11 ± 0.015 b	0.63 ± 0.29 a	0.81 ± 0.002 a
*Total*		0.21 ± 0.02 b	0.95 ± 0.03 a	1.09 ± 0.07 a
***Pyrazines***				
2-methylpyrazine	Roasted, burnt, sweet	0.19 ± 0.004 b	0.32 ± 0.065 a	0.30 ± 0.004 a
2,5-dimethylpyrazine	Crust-like, popcorn	0.09 ± 0.01 b	0.14 ± 0.041 ab	0.20 ± 0.007 a
2,6-dimethylpyrazine	Roasted	0.06 ± 0 c	0.12 ±0.051 b	0.21 ± 0.009 a
2-ethyl-6-methylpyrazine	Nutty	0.03 ± 0.007 b	0.15 ± 0.066 a	0.17 ± 0.004 a
2-ethyl-5-methylpyrazine	Baked	0.03 ± 0.017 b	0.07 ± 0.005 a	0.06 ± 0.009 a
2-ethyl-3-methylpyrazine	Nutty, roasted, sweety	0.08 ± 0.011 b	0.12 ± 0.016 a	0.11 ± 0.001 a
3-ethyl-2,5-dimethylpyrazine	Baked, earthy, potato-like	0.02 ± 0.001 b	0.08 ± 0.020 a	0.09 ± 0.001 a
Pyrazinamide	n.f.	0.02 ± 0.003 b	0.03 ± 0.014 ab	0.04 ± 0.001 a
*Total*		0.62 ± 0.01 b	1.17 ± 0.03 a	1.27 ± 0.07 a
***Pyrrolines***				
2-acetyl-1-pyrroline	Cracker-like	0.02 ± 0.010 b	0.09 ± 0.040 a	0.10± 0.013 a
3-hydroxy-2-methyl-4-pyrone (maltol)	Caramel, sweet	0.04 ± 0.014 b	0.22 ± 0.15 a	0.32 ± 0.047 a
*Total*		0.06 ± 0.01 b	0.31 ± 0.08 a	0.42 ± 0.03 a

^1^ n.d., not detected. ^2^ n.f., not found. Data are mean values ± standard deviation. a–c, Values in the same row with different letters differ significantly (*p* < 0.05).

## Supplementary Materials

The following are available online at https://www.mdpi.com/article/10.3390/foods10071489/s1, Table S1: Recipes for control and sourdough breads. CWB, control wheat bread started with baker’s yeast; SB-SL SWB EPS NEG, bread containing wheat flour added with 30% (*w/w*) sprouted barley–sprouted lentil (30:70%) sourdough fermented by Leuconostoc pseudomesenteroides DSM 20193; SB-SL SWB EPS POS, bread containing wheat flour added to 30% (*w/w*) dextran-containing sprouted barley–sprouted lentil (30:70% *w/w*) sourdough fermented by L. pseudomesenteroides DSM 20193. The strain was inoculated at ca. 7 log cfu/g and sourdough fermented at 20 °C for 24 h. Doughs for bread making had DY 162. Table S2: Acidity (pH) and viscosity (Pa s^−1^) values for doughs without sucrose addition (EPS NEG) and sucrose-supplemented (EPS POS) (16% of flour weight) doughs obtained from barley (B), sprouted barley (SB) and blends of sprouted barley with native lentil (SB-L) or sprouted lentil (SB-SL) flours at different ratios (60:40 and 70:30 ratios), before (T0 h) and after (T24 h) fermentation at 20 °C and 25 °C by *Lactobacillus plantarum* DPPMAB24W (B24W) as non EPS producing control or with the selected dextran-producing strains *Weissella confusa* SLA4 (SLA4), *Weissella paramesenteroides* SLA5 (SLA5), *Leuconostoc pseudomesenteroides* DSM 20193 (20193) and *Weissella confusa* DSM 20194 (20194). Doughs prepared without a starter and without the addition of sucrose were used as control (CT).
