# Supplementary material for: Bioprocessing of Barley and Lentil Grains to Obtain In Situ Synthesis of Exopolysaccharides and Composite Wheat Bread with Improved Texture and Health Properties
Source: Foods. 2021 Jun 27;10(7):1489. doi: 10.3390/foods10071489 (PMC8306093; doi:10.3390/foods10071489)
Supplement: Supplementary file 1 [file foods-10-01489-s001.zip › foods-1252966-s001.pdf]

Table S1 Recipes for control and sourdough breads. CWB, control wheat bread started with baker's yeast; SB-SL SWB EPS NEG, bread containing wheat flour added with 30% (w/w) sprouted barley-sprouted lentil (30:70%) sourdough fermented by *Leuconostoc pseudomesenteroides* DSM 20193; SB-SL SWB EPS POS, bread containing wheat flour added of 30% (w/w) dextran-containing sprouted barley-sprouted lentil (30:70% w/w) sourdough fermented by *L. pseudomesenteroides* DSM 20193. The strain was inoculated at ca. 7 log cfu/g and sourdough fermented at 20 °C for 24 h. Doughs for bread making had DY 162.

**Recipes**		**CWB**		**SB-SL SWB EPS NEG**		**SB-SL WB EPS POS**	
		**% d.w.**[1]	**% f.w.**[2]	**% d.w.**	**% f.w.**	**% d.w.**	**% f.w.**
**Sourdough composition**	Sprouted barley flour			2.7	4.5	2.7	4.5
	Sprouted lentil flour			6.4	10.5	6.4	10.5
	Water			21.2	35	21.2	35
	Sucrose			1.5	2.4	1.5	2.4
				**Bread**			
**Sourdough**		-	-	30.3	49	30.3	49
**Wheat flour**		61.7	100	51.7	85	51.7	85
**Water**		38.3	62	17.1	28	17.1	28
**Fresh yeast**		0.7	1.1	0.7	1.1	0.7	1.1
**Salt**		1.1	1.8	1.1	1.8	1.1	1.8
**Flour Sum**		61.7	100.0	61.7	100.0	61.7	100.0
**Water sum**		38.3	62.0	38.3	62.0	38.3	62.0

[1]d.w., dough weight

[2]f.w., flour weight

Table S2 Acidity (pH) and viscosity (Pa s^{-1}) values for doughs without sucrose addition (EPS NEG) and sucrose-supplemented (EPS POS) (16% of flour weight) obtained from native barley (B), sprouted barley (SB) and blends of sprouted barley with native lentil (SB-L) or sprouted lentil (SB-SL) flours at different ratio (60:40 and 70:30 ratios), before (T0 h) and after (T24 h) fermentation at 20°C and 25°C by *Lactobacillus plantarum* DPPMAB24W (B24W) as non EPS producing strain or with the selected dextran-producing strains *Weissella confusa* SLA4 (SLA4), *Weissella paramesenteroides* SLA5 (SLA5), *Leuconostoc pseudomesenteroides* DSM 20193 (20193) and *Weissella confusa* DSM 20194 (20194). Doughs prepared without starter and without the addition of sucrose were used as control (CT).

DY[1]	Sucrose	Dough Code	Fermentation at 20 °C				Fermentation at 25 °C			
			T0 h		T24 h		T0 h		T24 h	
			pH	Viscosity	pH	Viscosity	pH	Viscosity	pH	Viscosity
			**Native barley sourdough**							
500	-	B_CT	5.81 ± 0.14b	0.19 ± 0.02b	4.42 ± 0.18b	0.21 ± 0.13b	5.8 ± 0.04c	0.17 ± 0.02c	4.27 ± 0.16b	0.19 ± 0.06b
	+	B-B24W	5.84 ± 0.15b	0.16 ± 0.07c	3.66 ± 0.24c	0.23 ± 0.15b	5.85 ± 0.1c	0.21 ± 0.08b	3.56 ± 0.17c	0.23 ± 0.1b
	+	B-SLA4_EPS POS	5.85 ± 0.09b	0.14 ± 0.1c	3.74 ± 0.25c	0.3 ± 0.16b	5.83 ± 0.14c	0.16 ± 0.1c	3.63 ± 0.16c	0.39 ± 0.13b
	-	B-SLA4_EPS NEG	5.81 ± 0.08b	0.13 ± 0.12c	3.7 ± 0.26c	0.32 ± 0.16b	5.79 ± 0.06c	0.15 ± 0.12c	3.68 ± 0.01c	0.37 ± 0.14b
	+	B-SLA5_EPS POS	5.83 ± 0.11b	0.15 ± 0.05c	3.91 ± 0.08c	0.3 ± 0.16b	5.81 ± 0.17c	0.16 ± 0.13c	3.74 ± 0.19c	0.43 ± 0.02b
	-	B-SLA5_EPS NEG	5.75 ± 0.12b	0.12 ± 0.15c	3.88 ± 0.05c	0.33 ± 0.03b	5.86 ± 0.19c	0.14 ± 0.11c	3.82 ± 0.2c	0.35 ± 0.15b
	+	B-DSM20193_EPS POS	5.87 ± 0.03b	0.14 ± 0.02c	3.9 ± 0.19c	0.35 ± 0.17b	5.84 ± 0.2c	0.17 ± 0.15c	3.83 ± 0.21c	0.39 ± 0.15b
	-	B-DSM20193_EPS NEG	5.77 ± 0.05b	0.11 ± 0.16c	3.97 ± 0.3c	0.22 ± 0.34b	5.81 ± 0.21c	0.13 ± 0.01c	3.88 ± 0.08b	0.31 ± 0.36b
	+	B-DSM20194_EPS POS	5.81 ± 0.04b	0.15 ± 0.16c	3.95 ± 0.03c	0.3 ± 0.14b	5.81 ± 0.22c	0.14 ± 0.16c	3.62 ± 0.22c	0.39 ± 0.36b
	-	B-DSM20194_EPS NEG	5.9 ± 0.15b	0.16 ± 0.17c	3.91 ± 0.24c	0.32 ± 0.34b	5.78 ± 0.08c	0.17 ± 0.17c	3.6 ± 0.02c	0.43 ± 0.14b
			**Sprouted barley sourdough**							
500	-	SB_CT	5.83 ± 0.06b	0.11 ± 0.01c	4.01 ± 0.31c	0.22 ± 0.33b	5.83 ± 0.22c	0.13 ± 0.03c	3.82 ± 0.03c	0.35 ± 0.44b
	+	SB-B24W	5.84 ± 0.03b	0.13 ± 0.16c	3.62 ± 0.35c	0.26 ± 0.03b	5.84 ± 0.22c	0.14 ± 0.17c	3.51 ± 0.24c	0.37 ± 0.05b
	+	SB-SLA4_EPS POS	5.81 ± 0.15b	0.16 ± 0.16c	3.75 ± 0.34c	0.32 ± 0.32b	5.82 ± 0.22c	0.18 ± 0.16b	3.73 ± 0.22c	0.39 ± 0.44b

	-	SB-SLA4_EPS NEG	5.83 ± 0.15b	0.14 ± 0.01c	3.77 ± 0.33c	0.28 ± 0.32b	5.84 ± 0.21c	0.18 ± 0.16b	3.76 ± 0.22c	0.43 ± 0.01b
	+	SB-SLA5_EPS POS	5.8 ± 0.14b	0.13 ± 0.15c	3.72 ± 0.32c	0.26 ± 0.31b	5.81 ± 0.07c	0.14 ± 0.05c	3.61 ± 0.21c	0.35 ± 0.43b
	-	SB-SLA5_EPS NEG	5.75 ± 0.14b	0.15 ± 0.14c	3.74 ± 0.3c	0.3 ± 0.05b	5.77 ± 0.2c	0.15 ± 0.15c	3.68 ± 0.2c	0.33 ± 0.23b
	+	SB-DSM20193_EPS POS	5.85 ± 0.11b	0.16 ± 0.13c	3.71 ± 0.28c	0.63 ± 0.29b	5.86 ± 0.18c	0.17 ± 0.14c	3.72 ± 0.19c	0.39 ± 0.42b
	-	SB-DSM20193_EPS NEG	5.83 ± 0.15b	0.13 ± 0.11c	3.83 ± 0.26c	0.33 ± 0.41b	5.84 ± 0.16c	0.12 ± 0.13c	3.78 ± 0.17c	0.31 ± 0.04b
	+	SB-DSM20194_EPS POS	5.87 ± 0.15b	0.12 ± 0.09c	3.78 ± 0.24c	0.73 ± 0.01b	5.83 ± 0.15c	0.12 ± 0.11c	3.67 ± 0.16c	0.29 ± 0.48b
	-	SB-DSM20194_EPS NEG	5.78 ± 0.07b	0.19 ± 0.06	3.65 ± 0.22c	0.45 ± 0.4b	5.71 ± 0.12c	0.14 ± 0.08c	3.78 ± 0.14c	0.35 ± 0.51b
		**Sprouted barley-sprouted lentil sourdough (40:60 ratio)**								
	-	SB-SL_CT	6.23 ± 0.16a	0.45 ± 0.02a	4.75 ± 0.17a	0.63 ± 0.39b	6.24 ± 0.02b	0.48 ± 0.02a	4.31 ± 0a	0.74 ± 0.11b
	+	SB-SL-B24W	6.26 ± 0.16a	0.47 ± 0.03a	4.21 ± 0.16b	0.71 ± 0.39b	6.26 ± 0.03b	0.45 ± 0.02b	3.83 ± 0.12b	0.79 ± 0.51b
	+	SB-SL-SLA5_EPS POS	6.2 ± 0.18a	0.49 ± 0.03a	4.23 ± 0.16b	0.51 ± 0.29b	6.2 ± 0.03b	0.49 ± 0.03a	4.06 ± 0.01b	0.61 ± 0.51b
	-	SB-SL-SLA5_EPS NEG	6.21 ± 0.01a	0.45 ± 0a	4.37 ± 0.16b	0.48 ± 0.09b	6.23 ± 0.02b	0.47 ± 0a	4.09 ± 0.12b	0.64 ± 0.02b
**333**	+	SB-SL-SLA4_EPS POS	6.24 ± 0.04a	0.46 ± 0.03a	4.24 ± 0.16b	0.53 ± 0.39b	6.21 ± 0.02b	0.48 ± 0.03a	4.12 ± 0.12b	0.5 ± 0.5b
	-	SB-SL-SLA4_EPS NEG	6.27 ± 0.08a	0.48 ± 0a	4.28 ± 0.16b	0.55 ± 0.31b	6.23 ± 0.02b	0.46 ± 0.03a	4.16 ± 0b	0.65 ± 0.04b
	+	SB-SL-DSM20193_EPS POS	6.21 ± 0.09a	0.45 ± 0.03a	4.2 ± 0.16b	1.77 ± 0.39a	6.23 ± 0.02b	0.47 ± 0a	4 ± 0.12b	1.93 ± 0.48a

	-	SB-SL-DSM20193_EPS NEG	6.19 ± 0.12a	0.48 ± 0.01a	4.23 ± 0.05b	0.6 ± 0.03b	6.28 ± 0.02a	0.48 ± 0.03a	4.04 ± 0.02b	0.49 ± 0.02b
	+	SB-SL-DSM20194_EPS POS	6.22 ± 0.13a	0.47 ± 0.03a	4.21 ± 0b	0.63 ± 0.43b	6.21 ± 0.02b	0.51 ± 0.03a	4.11 ± 0.12b	1.72 ± 0.48a
	-	SB-SL-DSM20194_EPS NEG	6.18 ± 0.01a	0.45 ± 0.02a	4.33 ± 0.16b	0.51 ± 0.43b	6.25 ± 0.02b	0.47 ± 0.03a	4.14 ± 0.03b	0.63 ± 0.03b
**350**	-	SB-SL_CT	6.29 ± 0.05a	0.39 ± 0.02b	4.74 ± 0.01a	0.44 ± 0.33b	6.25 ± 0.02b	0.43 ± 0b	4.31 ± 0.12a	0.84 ± 0.5b
	+	SB-SL-B24W	6.31 ± 0.01a	0.42 ± 0.03b	4.24 ± 0.12b	0.58 ± 0.44b	6.26 ± 0.02b	0.42 ± 0.05b	3.83 ± 0.08b	0.79 ± 0.05b
	+	SB-SL-SLA5_EPS POS	6.25 ± 0.02a	0.41 ± 0.05b	4.24 ± 0.15b	0.76 ± 0.14b	6.23 ± 0.02b	0.47 ± 0.01a	4.06 ± 0.14b	0.95 ± 0.56b
	-	SB-SL-SLA5_EPS NEG	6.27 ± 0.01a	0.45 ± 0.05a	4.36 ± 0.15b	0.47 ± 0.47b	6.24 ± 0.02b	0.5 ± 0.06a	4.09 ± 0.15b	0.64 ± 0.58b
	+	SB-SL-SLA4_EPS POS	6.3 ± 0.16a	0.46 ± 0.06a	4.25 ± 0.14b	0.59 ± 0.39b	6.21 ± 0.02b	0.44 ± 0.07b	4.12 ± 0.15b	0.84 ± 0.03b
	-	SB-SL-SLA4_EPS NEG	6.33 ± 0.16a	0.45 ± 0a	4.27 ± 0.14b	0.48 ± 0.47b	6.23 ± 0.02b	0.51 ± 0.07a	4.16 ± 0.16b	0.65 ± 0.59b
	+	SB-SL-DSM20193_EPS POS	6.26 ± 0.16a	0.41 ± 0.05b	4.21 ± 0.14b	1.89 ± 0.47a	6.22 ± 0.02b	0.49 ± 0a	4 ± 0.16b	1.94 ± 0.59a
	-	SB-SL-DSM20193_EPS NEG	6.24 ± 0.01a	0.44 ± 0.05a	4.25 ± 0b	0.59 ± 0.26b	6.25 ± 0.02b	0.48 ± 0.06a	4.04 ± 0.01b	0.53 ± 0.49b
	+	SB-SL-DSM20194_EPS POS	6.28 ± 0.16a	0.43 ± 0.04b	4.22 ± 0.15b	0.84 ± 0.56b	6.25 ± 0.02b	0.44 ± 0.05b	4.11 ± 0.17b	1.82 ± 0.29a
	-	SB-SL-DSM20194_EPS NEG	6.24 ± 0.08a	0.42 ± 0.03b	4.32 ± 0.02b	0.53 ± 0.56b	6.22 ± 0.02b	0.42 ± 0.04b	4.14 ± 0.17b	0.66 ± 0.32b
**400**	-	SB-SL_CT	6.28 ± 0.16a	0.35 ± 0.02b	4.6 ± 0.15a	0.3 ± 0.47b	6.2 ± 0.03b	0.36 ± 0.01b	4.44 ± 0.06a	0.37 ± 0.34b
	+	SB-SL-B24W	6.22 ± 0.11a	0.32 ± 0.04b	4.02 ± 0.17c	0.33 ± 0.56b	6.22 ± 0b	0.38 ± 0.05b	3.91 ± 0.18b	0.39 ± 0.01b

	+	SB-SL-SLA5_EPS POS	6.2 ± 0.16a	0.33 ± 0.06b	4.23 ± 0.15b	0.37 ± 0.51b	6.21 ± 0.02b	0.32 ± 0.06b	3.94 ± 0.19b	0.41 ± 0.36b
	-	SB-SL-SLA5_EPS NEG	6.22 ± 0.12a	0.31 ± 0.06b	4.26 ± 0.15b	0.43 ± 0.56b	6.23 ± 0.02b	0.34 ± 0b	3.99 ± 0.2b	0.37 ± 0.27b
	+	SB-SL-SLA4_EPS POS	6.23 ± 0.16a	0.32 ± 0.06b	4.2 ± 0.02b	0.55 ± 0.06b	6.2 ± 0.02b	0.31 ± 0.06b	3.92 ± 0.21b	0.8 ± 0.17b
	-	SB-SL-SLA4_EPS NEG	6.25 ± 0.13a	0.37 ± 0b	4.24 ± 0.14b	0.49 ± 0.57b	6.24 ± 0b	0.36 ± 0.01b	3.95 ± 0.2b	0.57 ± 0.37b
	+	SB-SL-DSM20193_EPS POS	6.26 ± 0.01a	0.31 ± 0.01b	4.26 ± 0.13b	2.2 ± 0.27a	6.24 ± 0.02b	0.3 ± 0.06b	3.9 ± 0.19b	1.4 ± 0.38a
	-	SB-SL-DSM20193_EPS NEG	6.2 ± 0.15a	0.34 ± 0.06b	4.38 ± 0.12b	0.61 ± 0.37b	6.23 ± 0.02b	0.38 ± 0.06b	3.94 ± 0.07b	0.75 ± 0.29b
	+	SB-SL-DSM20194_EPS POS	6.24 ± 0.16a	0.34 ± 0.06b	4.23 ± 0.12b	0.5 ± 0.38b	6.27 ± 0.03a	0.34 ± 0.05b	3.92 ± 0.16b	0.9 ± 0.29b
	-	SB-SL-DSM20194_EPS NEG	6.29 ± 0.22a	0.38 ± 0.05b	4.36 ± 0.11b	0.42 ± 0.39b	6.27 ± 0.02a	0.3 ± 0.04b	4.01 ± 0.12b	0.38 ± 0.01b
**450**	-	SB-SL_CT	6.24 ± 0.17a	0.22 ± 0.03b	4.7 ± 0.11a	0.33 ± 0.39b	6.23 ± 0.02b	0.2 ± 0.03b	4.5 ± 0.1a	0.24 ± 0.02b
	+	SB-SL-B24W	6.2 ± 0.05a	0.2 ± 0.03b	4.36 ± 0.19b	0.38 ± 0.02b	6.2 ± 0b	0.22 ± 0b	4.25 ± 0.19b	0.28 ± 0.18b
	+	SB-SL-SLA5_EPS POS	6.22 ± 0.04a	0.26 ± 0.03b	4.41 ± 0.19b	0.4 ± 0.41b	6.25 ± 0.02b	0.25 ± 0.03b	4.22 ± 0.19b	0.28 ± 0.05b
	-	SB-SL-SLA5_EPS NEG	6.21 ± 0.01a	0.3 ± 0.04b	4.38 ± 0.2b	0.37 ± 0.28b	6.22 ± 0.02b	0.29 ± 0.04b	4.28 ± 0a	0.34 ± 0.26b
	+	SB-SL-SLA4_EPS POS	6.2 ± 0.08a	0.24 ± 0.03b	4.44 ± 0.19b	0.91 ± 0.42b	6.23 ± 0.02b	0.21 ± 0b	4.2 ± 0.02b	0.7 ± 0.27b
	-	SB-SL-SLA4_EPS NEG	6.26 ± 0.01a	0.25 ± 0.03b	4.54 ± 0.2b	0.33 ± 0.4b	6.24 ± 0.02b	0.25 ± 0.04b	4.18 ± 0.08b	0.33 ± 0.25b

	+	SB-SL-DSM20193_EPS POS	6.24 ± 0.03a	0.21 ± 0.03b	4.4 ± 0.2b	1.5 ± 0.01a	6.24 ± 0b	0.22 ± 0.04b	4.22 ± 0.07b	1 ± 0a
	-	SB-SL-DSM20193_EPS NEG	6.22 ± 0.05a	0.27 ± 0.03b	4.38 ± 0.2b	0.25 ± 0.39b	6.19 ± 0b	0.2 ± 0.01b	4.15 ± 0.04b	0.21 ± 0.05b
	+	SB-SL-DSM20194_EPS POS	6.26 ± 0.11a	0.23 ± 0.03b	4.43 ± 0.2b	0.7 ± 0.19b	6.21 ± 0.02b	0.24 ± 0.02b	4.21 ± 0.18b	0.3 ± 0b
	-	SB-SL-DSM20194_EPS NEG	6.24 ± 0.25a	0.25 ± 0b	4.35 ± 0.2b	0.42 ± 0.39b	6.23 ± 0.02b	0.27 ± 0.04b	4.24 ± 0.18b	0.32 ± 0.05b
500	-	SB-SL_CT	6.26 ± 0.04a	0.21 ± 0.03b	5 ± 0.2a	0.24 ± 0.4b	6.23 ± 0.02b	0.22 ± 0.03b	4.8 ± 0.01a	0.25 ± 0.03b
	+	SB-SL-B24W	6.2 ± 0.03a	0.18 ± 0.1b	4.39 ± 0.15b	0.2 ± 0.23b	6.21 ± 0.04b	0.21 ± 0.11b	4.41 ± 0.13a	0.24 ± 0.11b
	+	SB-SL-SLA5_EPS POS	6.23 ± 0.16a	0.19 ± 0.13b	4.63 ± 0.17a	0.21 ± 0.39b	6.24 ± 0.06b	0.16 ± 0.16c	4.22 ± 0.16b	0.18 ± 0.16b
	-	SB-SL-SLA5_EPS NEG	6.26 ± 0.16a	0.17 ± 0.14b	4.44 ± 0.16b	0.23 ± 0.37b	6.2 ± 0.06b	0.15 ± 0.19c	4.25 ± 0.19b	0.16 ± 0.19b
	+	SB-SL-SLA4_EPS POS	6.25 ± 0.16a	0.23 ± 0.15b	4.62 ± 0.01a	0.25 ± 0.13b	6.27 ± 0.06a	0.19 ± 0.19b	4.43 ± 0.23a	0.21 ± 0.19b
	-	SB-SL-SLA4_EPS NEG	6.22 ± 0.16a	0.25 ± 0.16b	4.56 ± 0.16b	0.23 ± 0.36b	6.24 ± 0.05b	0.17 ± 0.01c	4.36 ± 0.23a	0.19 ± 0b
	+	SB-SL-DSM20193_EPS POS	6.24 ± 0.16a	0.22 ± 0b	4.4 ± 0.15b	1.43 ± 0.34a	6.22 ± 0.05b	0.24 ± 0.18b	4.21 ± 0.26b	0.26 ± 0.19b
	-	SB-SL-DSM20193_EPS NEG	6.21 ± 0.16a	0.26 ± 0.16b	4.37 ± 0.15b	0.24 ± 0.49b	6.19 ± 0.05b	0.23 ± 0.17b	4.23 ± 0.01b	0.25 ± 0.48b
	+	SB-SL-DSM20194_EPS POS	6.21 ± 0.16a	0.18 ± 0.14b	4.41 ± 0.16b	0.75 ± 0.4b	6.21 ± 0.05b	0.19 ± 0.16b	4.42 ± 0.29a	0.21 ± 0.47b

	-	SB-SL-DSM20194_EPS NEG	6.22 ± 0.16a	0.25 ± 0.1b	4.39 ± 0.01b	0.27 ± 0.92b	6.25 ± 0.04b	0.22 ± 0.11b	4.31 ± 0.27a	0.24 ± 0.13b
**Sprouted barley-sprouted lentil sourdough (30:70 ratio)**										
**333**	-	SB-SL_CT	6.1 ± 0.03a	0.52 ± 0.03a	4.81 ± 0.17a	0.57 ± 0.9b	6.12 ± 0.02b	0.54 ± 0.03a	4.61 ± 0.26a	0.56 ± 0.5b
	+	SB-SL-B24W	6.11 ± 0.08a	0.51 ± 0.06a	4.22 ± 0.16b	0.56 ± 0.65b	6.1 ± 0.02b	0.59 ± 0.06a	4.04 ± 0.02b	0.61 ± 0.51b
	+	SB-SL-SLA5_EPS POS	6.14 ± 0.04a	0.5 ± 0.07a	4.41 ± 0.16b	0.53 ± 0.88b	6.13 ± 0.02b	0.6 ± 0.08a	3.94 ± 0.26b	0.63 ± 0.22b
	-	SB-SL-SLA5_EPS NEG	6.17 ± 0.01a	0.55 ± 0.08a	4.46 ± 0.16b	0.57 ± 0.92b	6.16 ± 0.02b	0.56 ± 0a	3.91 ± 0.26b	0.59 ± 0.43b
	+	SB-SL-SLA4_EPS POS	6.12 ± 0.01a	0.57 ± 0.09a	4.33 ± 0.16b	0.9 ± 0.93b	6.14 ± 0.02b	0.55 ± 0.09a	4 ± 0.26b	0.57 ± 0.05b
	-	SB-SL-SLA4_EPS NEG	6.15 ± 0.01a	0.58 ± 0.1a	4.38 ± 0.16b	0.64 ± 0.26b	6.15 ± 0.02b	0.58 ± 0.01a	3.76 ± 0.26c	0.62 ± 0.35b
	+	SB-SL-DSM20193_EPS POS	6.15 ± 0.02a	0.59 ± 0.09a	4.32 ± 0.1b	3.3 ± 0.93a	6.12 ± 0.02b	0.59 ± 0.09a	3.82 ± 0.26c	1.9 ± 0.56a
	-	SB-SL-DSM20193_EPS NEG	6.16 ± 0.15a	0.54 ± 0.08a	4.35 ± 0.16b	0.62 ± 0.89b	6.18 ± 0.02b	0.54 ± 0.08a	3.78 ± 0.14c	0.61 ± 0.17b
	+	SB-SL-DSM20194_EPS POS	6.13 ± 0.14a	0.52 ± 0.01a	4.23 ± 0.16b	1.9 ± 0.5a	6.13 ± 0.02b	0.52 ± 0.07a	4.2 ± 0.11b	1.67 ± 0.62a
	-	SB-SL-DSM20194_EPS NEG	6.16 ± 0.16a	0.57 ± 0.06a	4.41 ± 0b	0.61 ± 0.91b	6.16 ± 0.02b	0.57 ± 0.06a	4.18 ± 0.26b	0.61 ± 0.66b
**350**	-	SB-SL_CT	6.11 ± 0.18a	0.38 ± 0.02b	4.79 ± 0a	0.43 ± 0.65b	6.12 ± 0.02b	0.38 ± 0.03b	4.61 ± 0.26a	0.41 ± 0.38b
	+	SB-SL-B24W	6.1 ± 0.21a	0.41 ± 0.05b	4.21 ± 0.19b	0.53 ± 0.93b	6.11 ± 0.02b	0.41 ± 0.05b	4.04 ± 0.21b	0.45 ± 0.39b
	+	SB-SL-SLA5_EPS POS	6.11 ± 0.22a	0.4 ± 0.06b	4.38 ± 0.08b	0.45 ± 0.71b	6.12 ± 0.02b	0.42 ± 0.06b	3.94 ± 0.23b	0.48 ± 0.7b
	-	SB-SL-SLA5_EPS NEG	6.16 ± 0.15a	0.39 ± 0b	4.45 ± 0.21b	0.41 ± 0.95b	6.14 ± 0.02b	0.38 ± 0.07b	3.91 ± 0.23b	0.39 ± 0.71b

	+	SB-SL-SLA4_EPS POS	6.12 ± 0.19a	0.34 ± 0b	4.32 ± 0.2b	0.93 ± 0.96b	6.13 ± 0.02b	0.35 ± 0.01b	4 ± 0b	0.53 ± 0.34b
	-	SB-SL-SLA4_EPS NEG	6.15 ± 0.09a	0.36 ± 0.07b	4.37 ± 0.21b	0.48 ± 0.97b	6.14 ± 0.03b	0.39 ± 0.07b	3.76 ± 0.23c	0.41 ± 0.44b
	+	SB-SL-DSM20193_EPS POS	6.14 ± 0.09a	0.39 ± 0b	4.31 ± 0.02b	3.15 ± 0.98a	6.11 ± 0.03b	0.37 ± 0.02b	3.82 ± 0.21c	2.15 ± 0.73a
	-	SB-SL-DSM20193_EPS NEG	6.15 ± 0.08a	0.41 ± 0.07b	4.34 ± 0.22b	0.42 ± 0.57b	6.17 ± 0.02b	0.43 ± 0.07b	3.78 ± 0.2c	0.48 ± 0.58b
	+	SB-SL-DSM20194_EPS POS	6.12 ± 0.05a	0.35 ± 0.05b	4.22 ± 0.22b	1.98 ± 0.47a	6.13 ± 0.02b	0.39 ± 0.05b	4.2 ± 0.18b	1.89 ± 0.19a
	-	SB-SL-DSM20194_EPS NEG	6.15 ± 0.01a	0.39 ± 0.05b	4.42 ± 0.01b	0.43 ± 0.37b	6.16 ± 0.02b	0.36 ± 0.04b	4.18 ± 0.17b	0.42 ± 0.45b
**400**	-	SB-SL_CT	6.12 ± 0.02a	0.25 ± 0.02b	4.9 ± 0.21a	0.33 ± 0.38b	6.1 ± 0.02b	0.26 ± 0.02b	4.41 ± 0a	0.14 ± 0.25b
	+	SB-SL-B24W	6.13 ± 0.01a	0.26 ± 0.02b	4.13 ± 0.06b	0.31 ± 0.08b	6.12 ± 0b	0.24 ± 0b	3.72 ± 0.17c	0.29 ± 0.01b
	+	SB-SL-SLA5_EPS POS	6.18 ± 0.03a	0.23 ± 0.02b	4.31 ± 0.14b	0.28 ± 0.39b	6.14 ± 0.02b	0.26 ± 0.02b	4 ± 0.14b	0.28 ± 0.44b
	-	SB-SL-SLA5_EPS NEG	6.15 ± 0.02a	0.25 ± 0.02b	4.36 ± 0.15b	0.33 ± 0.39b	6.13 ± 0.01b	0.28 ± 0.02b	3.98 ± 0.14b	0.28 ± 0.23b
	+	SB-SL-SLA4_EPS POS	6.16 ± 0.01a	0.28 ± 0.03b	4.23 ± 0.03b	0.32 ± 0.4b	6.18 ± 0.04b	0.27 ± 0.03b	4.04 ± 0.04b	0.31 ± 0.44b
	-	SB-SL-SLA4_EPS NEG	6.19 ± 0.07a	0.29 ± 0.04b	4.18 ± 0.14b	0.31 ± 0.42b	6.14 ± 0.02b	0.29 ± 0.04b	4.01 ± 0.05b	0.36 ± 0.17b
	+	SB-SL-DSM20193_EPS POS	6.12 ± 0.11a	0.22 ± 0.01b	4.34 ± 0.12b	1.3 ± 0.03a	6.16 ± 0.05b	0.23 ± 0.04b	4.12 ± 0.14b	1.44 ± 0.45a
	-	SB-SL-DSM20193_EPS NEG	6.17 ± 0.12a	0.26 ± 0.04b	4.37 ± 0.12b	0.96 ± 0.43b	6.15 ± 0.06b	0.25 ± 0.05b	4.08 ± 0.15b	0.28 ± 0.31b

	+	SB-SL-DSM20194_EPS POS	6.13 ± 0.13a	0.27 ± 0.04b	4.41 ± 0.12b	0.92 ± 0.34b	6.13 ± 0.02b	0.28 ± 0.04b	4.11 ± 0.16b	1.25 ± 0.31a
	-	SB-SL-DSM20194_EPS NEG	6.1 ± 0.13a	0.25 ± 0.03b	4.38 ± 0.12b	0.27 ± 0.42b	6.15 ± 0.02b	0.26 ± 0b	3.99 ± 0.17b	0.31 ± 0.05b
**450**	-	SB-SL_CT	6.17 ± 0.21a	0.24 ± 0.03b	4.72 ± 0.12a	0.25 ± 0.31b	6.16 ± 0.07b	0.26 ± 0.04b	4.43 ± 0.17a	0.34 ± 0.06b
	+	SB-SL-B24W	6.09 ± 0.15a	0.25 ± 0.03b	4.31 ± 0.21b	0.27 ± 0.43b	6.14 ± 0.03b	0.25 ± 0.03b	3.92 ± 0.23b	0.38 ± 0b
	+	SB-SL-SLA5_EPS POS	6.1 ± 0.15a	0.21 ± 0.02b	4.5 ± 0.2b	0.23 ± 0.43b	6.11 ± 0.06b	0.23 ± 0.03b	4 ± 0.23b	0.33 ± 0.04b
	-	SB-SL-SLA5_EPS NEG	6.09 ± 0.02a	0.19 ± 0b	4.48 ± 0.21b	0.22 ± 0.21b	6.12 ± 0.03b	0.17 ± 0.03c	3.98 ± 0.24b	0.23 ± 0.03b
	+	SB-SL-SLA4_EPS POS	6.14 ± 0.02a	0.17 ± 0.03b	4.31 ± 0.21b	0.97 ± 0.44b	6.18 ± 0.03b	0.16 ± 0.03c	4.01 ± 0.02b	0.26 ± 0.01b
	-	SB-SL-SLA4_EPS NEG	6.13 ± 0.15a	0.15 ± 0.03b	4.4 ± 0.2b	0.18 ± 0.44b	6.15 ± 0.02b	0.17 ± 0.03c	4.05 ± 0.24b	0.21 ± 0.04b
	+	SB-SL-DSM20193_EPS POS	6.11 ± 0.01a	0.19 ± 0.04b	4.47 ± 0.2b	1.33 ± 0.44a	6.14 ± 0b	0.18 ± 0.04b	3.91 ± 0.24b	0.28 ± 0b
	-	SB-SL-DSM20193_EPS NEG	6.1 ± 0.15a	0.21 ± 0.04b	4.38 ± 0.2b	0.23 ± 0.29b	6.12 ± 0b	0.22 ± 0.04b	3.89 ± 0.23b	0.25 ± 0.38b
	+	SB-SL-DSM20194_EPS POS	6.12 ± 0.03a	0.23 ± 0.04b	4.44 ± 0.2b	0.91 ± 0.44b	6.1 ± 0.03b	0.25 ± 0.04b	3.8 ± 0.21c	0.27 ± 0.38b
	-	SB-SL-DSM20194_EPS NEG	6.15 ± 0.02a	0.17 ± 0b	4.39 ± 0.2b	0.19 ± 0.24b	6.12 ± 0.02b	0.19 ± 0.02b	3.88 ± 0.23b	0.22 ± 0.06b
**500**	-	SB-SL_CT	6.14 ± 0.15a	0.19 ± 0.02b	5.03 ± 0.19a	0.21 ± 0.44b	6.17 ± 0.01b	0.18 ± 0b	4.64 ± 0.22a	0.21 ± 0.38b
	+	SB-SL-B24W	6.12 ± 0.15a	0.16 ± 0.02b	4.42 ± 0.09b	0.19 ± 0.16b	6.14 ± 0.02b	0.17 ± 0.02c	3.92 ± 0.11b	0.23 ± 0.4b
	+	SB-SL-SLA5_EPS POS	6.11 ± 0.14a	0.14 ± 0.14b	4.63 ± 0.1a	0.17 ± 0.45b	6.18 ± 0.04b	0.15 ± 0.11c	4.13 ± 0.13b	0.18 ± 0.13b

	-	SB-SL-SLA5_EPS NEG	6.1 ± 0.16a	0.13 ± 0.18b	4.43 ± 0.1b	0.15 ± 0.43b	6.16 ± 0.05b	0.14 ± 0.15c	4.08 ± 0.15b	0.17 ± 0.44b
	+	SB-SL-SLA4_EPS POS	6.13 ± 0.17a	0.15 ± 0.19b	4.43 ± 0.11b	0.97 ± 0.27b	6.15 ± 0.01b	0.16 ± 0.16c	4.12 ± 0.15b	0.19 ± 0.33b
	-	SB-SL-SLA4_EPS NEG	6.14 ± 0.15a	0.12 ± 0.21b	4.36 ± 0b	0.18 ± 0.19b	6.17 ± 0.07b	0.13 ± 0.17c	4.09 ± 0.15b	0.16 ± 0.41b
	+	SB-SL-DSM20193_EPS POS	6.12 ± 0.02a	0.14 ± 0.21b	4.61 ± 0.1a	1.32 ± 0.35a	6.14 ± 0.08b	0.15 ± 0.18c	4.04 ± 0.15b	1.4 ± 0.38a
	-	SB-SL-DSM20193_EPS NEG	6.09 ± 0.03a	0.11 ± 0.2b	4.58 ± 0.1b	0.16 ± 0.23b	6.19 ± 0.07b	0.13 ± 0.11c	4.11 ± 0.16b	0.18 ± 0.24b
	+	SB-SL-DSM20194_EPS POS	6.15 ± 0.03a	0.15 ± 0.17b	4.47 ± 0.11b	0.9 ± 0.44b	6.12 ± 0.01b	0.17 ± 0.15c	3.81 ± 0c	0.19 ± 0.28b
	-	SB-SL-DSM20194_EPS NEG	6.07 ± 0.15a	0.16 ± 0.13b	4.49 ± 0.12b	0.21 ± 0.23b	6.15 ± 0.05b	0.18 ± 0.12b	3.98 ± 0.13b	0.24 ± 0.24b
**Sprouted barley-native lentil sourdough (40:60 ratio)**										
	-	SB-L_CT	6.27 ± 0.16a	0.54 ± 0.02a	4.65 ± 0.12a	0.63 ± 0.39b	6.25 ± 0.02b	0.49 ± 0.04a	4.31 ± 0.12a	0.74 ± 0.37b
	+	SB-L -B24W	6.26 ± 0.23a	0.56 ± 0.05a	4.41 ± 0.14b	0.71 ± 0.34b	6.27 ± 0.03b	0.47 ± 0.05a	3.83 ± 0.12b	0.79 ± 0.18b
	+	SB-L -SLA5_EPS POS	6.25 ± 0.22a	0..54 ± 0.06a	4.33 ± 0.15b	0.51 ± 0.22b	6.28 ± 0.01a	0.44 ± 0.05b	4.06 ± 0.02b	0.61 ± 0.02b
**333**	-	SB-L -SLA5_EPS NEG	6.24 ± 0.16a	0.51 ± 0.06a	4.57 ± 0.16b	0.48 ± 0.39b	6.33 ± 0.04a	0.51 ± 0.05a	4.09 ± 0.12b	0.64 ± 0.37b
	+	SB-L -SLA4_EPS POS	6.3 ± 0.18a	0.52 ± 0a	4.54 ± 0.16b	0.53 ± 0.34b	6.31 ± 0.04a	0.58 ± 0.05a	4.12 ± 0.01b	0.5 ± 0.27b
	-	SB-L -SLA4_EPS NEG	6.29 ± 0.14a	0.53 ± 0.06a	4.48 ± 0.15b	0.55 ± 0.39b	6.29 ± 0.04b	0.52 ± 0a	4.16 ± 0.12b	0.65 ± 0.35b
	+	SB-L -DSM20193_EPS POS	6.26 ± 0.16a	0.51 ± 0a	4.3 ± 0.02b	1.77 ± 0.39a	6.28 ± 0b	0.51 ± 0.04a	4 ± 0.12b	1.13 ± 0.03a

	-	SB-L - DSM20193_EPS NEG	6.21 ± 0.18a	0.52 ± 0.06a	4.33 ± 0.16b	0.6 ± 0.13b	6.25 ± 0.04b	0.54 ± 0.04a	4.04 ± 0.03b	0.49 ± 0.48b
	+	SB-L - DSM20194_EPS POS	6.22 ± 0.08a	0.55 ± 0.06a	4.31 ± 0.16b	0.63 ± 0.43b	6.28 ± 0.03b	0.51 ± 0.03a	4.11 ± 0.12b	1.72 ± 0.18a
	-	SB-L - DSM20194_EPS NEG	6.24 ± 0.08a	0.55 ± 0.04a	4.43 ± 0.03b	0.51 ± 0.41b	6.29 ± 0.03b	0.5 ± 0.03a	4.14 ± 0.12b	0.63 ± 0.2b
**350**	-	SB-L_CT	6.28 ± 0.16a	0.39 ± 0.02b	4.73 ± 0.16a	0.44 ± 0.22b	6.23 ± 0.02b	0.43 ± 0.03b	4.29 ± 0.12a	0.84 ± 0.5b
	+	SB-L -B24W	6.3 ± 0.01a	0.42 ± 0.03b	4.23 ± 0.12b	0.58 ± 0.44b	6.23 ± 0.02b	0.42 ± 0.02b	3.81 ± 0.15c	0.79 ± 0.53b
	+	SB-L -SLA5_EPS POS	6.24 ± 0.02a	0.41 ± 0.02b	4.23 ± 0.14b	0.76 ± 0.16b	6.21 ± 0.02b	0.47 ± 0.05a	4.04 ± 0.14b	0.95 ± 0.26b
	-	SB-L -SLA5_EPS NEG	6.26 ± 0.01a	0.45 ± 0.05a	4.35 ± 0.01b	0.47 ± 0.47b	6.22 ± 0.02b	0.5 ± 0.06a	4.07 ± 0.15b	0.64 ± 0.57b
	+	SB-L -SLA4_EPS POS	6.29 ± 0.01a	0.46 ± 0.06a	4.24 ± 0.04b	0.59 ± 0.38b	6.19 ± 0.02b	0.44 ± 0.07b	4.1 ± 0.15b	0.84 ± 0.29b
	-	SB-L -SLA4_EPS NEG	6.32 ± 0.02a	0.45 ± 0.01a	4.26 ± 0.05b	0.48 ± 0.45b	6.21 ± 0.02b	0.51 ± 0a	4.14 ± 0.09b	0.65 ± 0.49b
	+	SB-L - DSM20193_EPS POS	6.25 ± 0.15a	0.41 ± 0.05b	4.2 ± 0.01b	1.89 ± 0.47a	6.2 ± 0.02b	0.49 ± 0.06a	3.98 ± 0.16b	1.93 ± 0.59a
	-	SB-L - DSM20193_EPS NEG	6.23 ± 0.11a	0.44 ± 0.05a	4.24 ± 0.06b	0.59 ± 0.51b	6.23 ± 0.02b	0.48 ± 0a	4.02 ± 0.16b	0.53 ± 0.19b
	+	SB-L - DSM20194_EPS POS	6.27 ± 0.12a	0.43 ± 0b	4.21 ± 0.09b	0.84 ± 0.52b	6.23 ± 0.02b	0.44 ± 0.05b	4.09 ± 0.17b	1.81 ± 0.49a
	-	SB-L - DSM20194_EPS NEG	6.23 ± 0.05a	0.42 ± 0.03b	4.31 ± 0.08b	0.53 ± 0.54b	6.2 ± 0.02b	0.42 ± 0.04b	4.12 ± 0.07b	0.66 ± 0.32b
**400**	-	SB-L_CT	6.27 ± 0.02a	0.35 ± 0.02b	4.59 ± 0.07a	0.3 ± 0.41b	6.18 ± 0.03b	0.36 ± 0.01b	4.42 ± 0.16a	0.37 ± 0.33b

	+	SB-L -B24W	6.21 ± 0.21a	0.32 ± 0.04b	4.01 ± 0.17c	0.33 ± 0.54b	6.2 ± 0.02b	0.38 ± 0.05b	3.89 ± 0.18b	0.39 ± 0.35b
	+	SB-L -SLA5_EPS POS	6.19 ± 0.13a	0.33 ± 0.06b	4.22 ± 0.02b	0.32 ± 0.51b	6.19 ± 0.02b	0.32 ± 0.06b	3.92 ± 0.19b	0.41 ± 0.08b
	-	SB-L -SLA5_EPS NEG	6.21 ± 0.16a	0.31 ± 0.06b	4.25 ± 0.15b	0.39 ± 0.53b	6.21 ± 0.02b	0.34 ± 0b	3.97 ± 0.04b	0.37 ± 0.37b
	+	SB-L -SLA4_EPS POS	6.22 ± 0.02a	0.32 ± 0b	4.19 ± 0.14b	0.48 ± 0.29b	6.18 ± 0.02b	0.31 ± 0.01b	3.9 ± 0.21b	0.8 ± 0.27b
	-	SB-L -SLA4_EPS NEG	6.24 ± 0.03a	0.37 ± 0.06b	4.23 ± 0.13b	0.56 ± 0.54b	6.22 ± 0.02b	0.36 ± 0.06b	3.93 ± 0.2b	0.57 ± 0.31b
	+	SB-L -DSM20193_EPS POS	6.25 ± 0.02a	0.31 ± 0b	4.25 ± 0.13b	2.11 ± 0.25a	6.22 ± 0.02b	0.3 ± 0.01b	3.88 ± 0.19b	1.39 ± 0.37a
	-	SB-L -DSM20193_EPS NEG	6.19 ± 0.01a	0.34 ± 0.06b	4.37 ± 0.12b	0.77 ± 0.38b	6.21 ± 0.02b	0.38 ± 0.06b	3.92 ± 0.17b	0.75 ± 0.29b
	+	SB-L -DSM20194_EPS POS	6.23 ± 0.02a	0.34 ± 0.01b	4.22 ± 0.12b	0.68 ± 0.39b	6.24 ± 0.03b	0.34 ± 0.05b	3.9 ± 0.15b	0.9 ± 0.29b
	-	SB-L -DSM20194_EPS NEG	6.28 ± 0.03a	0.38 ± 0.05b	4.35 ± 0.11b	0.47 ± 0.33b	6.24 ± 0.02b	0.3 ± 0.04b	3.99 ± 0.12b	0.38 ± 0.25b
**450**	-	SB-L_CT	6.23 ± 0.06a	0.22 ± 0.03b	4.69 ± 0a	0.27 ± 0.38b	6.21 ± 0.02b	0.2 ± 0.03b	4.48 ± 0.1a	0.24 ± 0.13b
	+	SB-L -B24W	6.19 ± 0.15a	0.2 ± 0b	4.35 ± 0.19b	0.38 ± 0.39b	6.18 ± 0.02b	0.22 ± 0.01b	4.23 ± 0.19b	0.28 ± 0.25b
	+	SB-L -SLA5_EPS POS	6.21 ± 0.18a	0.26 ± 0.03b	4.4 ± 0.19b	0.4 ± 0.4b	6.23 ± 0.02b	0.25 ± 0.03b	4.2 ± 0.19b	0.28 ± 0.25b
	-	SB-L -SLA5_EPS NEG	6.2 ± 0.19a	0.3 ± 0.04b	4.37 ± 0.2b	0.37 ± 0.21b	6.2 ± 0.02b	0.29 ± 0.04b	4.26 ± 0.19b	0.34 ± 0.11b
	+	SB-L -SLA4_EPS POS	6.19 ± 0.15a	0.24 ± 0.03b	4.43 ± 0.19b	0.91 ± 0.42b	6.21 ± 0.02b	0.21 ± 0.04b	4.18 ± 0.19b	0.7 ± 0.27b
	-	SB-L -SLA4_EPS NEG	6.25 ± 0.03a	0.25 ± 0b	4.53 ± 0.2b	0.33 ± 0.4b	6.22 ± 0.02b	0.25 ± 0.04b	4.16 ± 0.19b	0.33 ± 0.24b

	+	SB-L -DSM20193_EPS POS	6.23 ± 0.06a	0.21 ± 0.03b	4.39 ± 0.2b	1.5 ± 0.41a	6.22 ± 0b	0.22 ± 0b	4.2 ± 0.01b	1 ± 0.25a
	-	SB-L -DSM20193_EPS NEG	6.21 ± 0.01a	0.27 ± 0.03b	4.37 ± 0.01b	0.25 ± 0.43b	6.17 ± 0.01b	0.2 ± 0.04b	4.13 ± 0.19b	0.21 ± 0.05b
	+	SB-L -DSM20194_EPS POS	6.25 ± 0.16a	0.23 ± 0.01b	4.42 ± 0.03b	0.7 ± 0.41b	6.19 ± 0.02b	0.24 ± 0.04b	4.19 ± 0.18b	0.3 ± 0.01b
	-	SB-L -DSM20194_EPS NEG	6.23 ± 0.16a	0.25 ± 0.01b	4.34 ± 0.2b	0.42 ± 0.42b	6.21 ± 0.02b	0.27 ± 0.04b	4.22 ± 0.02b	0.32 ± 0.05b
**500**	-	SB-L_CT	6.25 ± 0.16a	0.21 ± 0.03b	4.99 ± 0.19a	0.24 ± 0.36b	6.21 ± 0.02b	0.22 ± 0.03b	4.78 ± 0.18a	0.25 ± 0.03b
	+	SB-L -B24W	6.19 ± 0.15a	0.18 ± 0.01b	4.38 ± 0.1b	0.2 ± 0.28b	6.19 ± 0.03b	0.21 ± 0b	4.39 ± 0.09a	0.24 ± 0.03b
	+	SB-L -SLA5_EPS POS	6.22 ± 0.16a	0.19 ± 0.03b	4.62 ± 0.11a	0.21 ± 0.08b	6.22 ± 0.03b	0.16 ± 0.03c	4.2 ± 0.09b	0.18 ± 0.04b
	-	SB-L -SLA5_EPS NEG	6.25 ± 0.16a	0.17 ± 0.1c	4.43 ± 0.08b	0.23 ± 0.47b	6.18 ± 0.05b	0.15 ± 0.11c	4.23 ± 0.08b	0.16 ± 0.13b
	+	SB-L -SLA4_EPS POS	6.24 ± 0.16a	0.23 ± 0.12b	4.61 ± 0.11a	0.25 ± 0.05b	6.24 ± 0.03b	0.19 ± 0.16b	4.41 ± 0.12a	0.21 ± 0.17b
	-	SB-L -SLA4_EPS NEG	6.21 ± 0.16a	0.25 ± 0.14b	4.55 ± 0.1b	0.23 ± 0.44b	6.22 ± 0.01b	0.17 ± 0.01c	4.34 ± 0.18a	0.19 ± 0.19b
	+	SB-L -DSM20193_EPS POS	6.23 ± 0.16a	0.22 ± 0.16b	4.39 ± 0.1b	1.56 ± 0.41a	6.2 ± 0.03b	0.24 ± 0.19b	4.19 ± 0.21b	0.26 ± 0.2b
	-	SB-L -DSM20193_EPS NEG	6.2 ± 0.15a	0.26 ± 0.15b	4.36 ± 0.12b	0.24 ± 0.17b	6.17 ± 0.03b	0.23 ± 0b	4.21 ± 0.23b	0.25 ± 0b
	+	SB-L -DSM20194_EPS POS	6.2 ± 0.15a	0.18 ± 0.14b	4.4 ± 0.04b	0.75 ± 0.14b	6.19 ± 0.03b	0.19 ± 0.17b	4.4 ± 0.25a	0.21 ± 0.18b

	-	SB-L -DSM20194_EPS NEG	6.21 ± 0.16a	0.25 ± 0.09b	4.38 ± 0.13b	0.27 ± 0.85b	6.23 ± 0.05b	0.22 ± 0.12b	4.29 ± 0.21a	0.24 ± 0.41b
		**Sprouted barley-native lentil sourdough (30:70 ratio)**								
333	-	SB-L_CT	6.28 ± 0.16a	0.48 ± 0.03a	4.61 ± 0.11a	0.57 ± 0.33b	6.26 ± 0.06b	0.51 ± 0.03a	4.31 ± 0.19a	0.56 ± 0.33b
	+	SB-L -B24W	6.25 ± 0.16a	0.5 ± 0.02a	4.38 ± 0.05b	0.56 ± 0.77b	6.2 ± 0.06b	0.59 ± 0.03a	4.04 ± 0.16b	0.61 ± 0.34b
	+	SB-L -SLA5_EPS POS	6.24 ± 0.16a	0.53 ± 0.05a	4.31 ± 0.17b	0.53 ± 0.18b	6.23 ± 0.07b	0.6 ± 0.06a	3.84 ± 0.27b	0.63 ± 0.36b
	-	SB-L -SLA5_EPS NEG	6.26 ± 0.16a	0.54 ± 0.01a	4.26 ± 0.18b	0.53 ± 0.07b	6.26 ± 0.08b	0.56 ± 0.02a	3.84 ± 0.26b	0.63 ± 0.18b
	+	SB-L -SLA4_EPS POS	6.28 ± 0.16a	0.49 ± 0.06a	4.23 ± 0.18b	0.57 ± 0.78b	6.24 ± 0.08b	0.55 ± 0.07a	3.91 ± 0.25b	0.64 ± 0.39b
	-	SB-L -SLA4_EPS NEG	6.29 ± 0.16a	0.52 ± 0.07a	4.28 ± 0.01b	0.64 ± 0.79b	6.21 ± 0.07b	0.52 ± 0.08a	3.76 ± 0.25c	0.62 ± 0.23b
	+	SB-L -DSM20193_EPS POS	6.25 ± 0.16a	0.51 ± 0.08a	4.22 ± 0.18b	2.9 ± 0.69a	6.12 ± 0.07b	0.52 ± 0a	3.92 ± 0.23b	1.65 ± 0.22a
	-	SB-L -DSM20193_EPS NEG	6.22 ± 0.05a	0.51 ± 0.08a	4.24 ± 0.17b	0.62 ± 0.19b	6.28 ± 0.07a	0.54 ± 0.08a	3.86 ± 0.25b	0.61 ± 0.12b
	+	SB-L -DSM20194_EPS POS	6.21 ± 0.06a	0.5 ± 0.07a	4.33 ± 0.16b	0.9 ± 0.73b	6.23 ± 0.04b	0.52 ± 0.07a	4.2 ± 0.26b	0.7 ± 0.5b
	-	SB-L -DSM20194_EPS NEG	6.16 ± 0.15a	0.57 ± 0.07a	4.31 ± 0.16b	0.61 ± 0.44b	6.12 ± 0.02b	0.57 ± 0.07a	4.18 ± 0.14b	0.61 ± 0.26b
350	-	SB-L_CT	6.1 ± 0.02a	0.38 ± 0.02b	4.78 ± 0.16a	0.43 ± 0.4b	6.1 ± 0.02b	0.38 ± 0.03b	4.59 ± 0.26a	0.41 ± 0.33b
	+	SB-L -B24W	6.09 ± 0.09a	0.41 ± 0.05b	4.2 ± 0.19b	0.53 ± 0.8b	6.09 ± 0.02b	0.41 ± 0.05b	4.02 ± 0.21b	0.33 ± 0.65b
	+	SB-L -SLA5_EPS POS	6.1 ± 0.09a	0.4 ± 0.06b	4.37 ± 0.2b	0.39 ± 0.47b	6.1 ± 0.02b	0.42 ± 0.06b	3.92 ± 0.08b	0.48 ± 0.15b

	-	SB-L -SLA5_EPS NEG	6.15 ± 0.05a	0.39 ± 0.07b	4.44 ± 0.01b	0.41 ± 0.82b	6.12 ± 0.02b	0.38 ± 0.07b	3.89 ± 0.23b	0.39 ± 0.66b
	+	SB-L -SLA4_EPS POS	6.19 ± 0.06a	0.34 ± 0.01b	4.31 ± 0.08b	0.86 ± 0.83b	6.11 ± 0.02b	0.35 ± 0.01b	3.98 ± 0.03b	0.53 ± 0.67b
	-	SB-L -SLA4_EPS NEG	6.14 ± 0.05a	0.36 ± 0.07b	4.36 ± 0.21b	0.43 ± 0.84b	6.12 ± 0.03b	0.39 ± 0.07b	3.74 ± 0.22c	0.41 ± 0.71b
	+	SB-L -DSM20193_EPS POS	6.11 ± 0.11a	0.39 ± 0.01b	4.34 ± 0.02b	2.67 ± 0.59a	6.09 ± 0.03b	0.37 ± 0b	3.8 ± 0.21c	1.95 ± 0.68a
	-	SB-L -DSM20193_EPS NEG	6.18 ± 0.15a	0.41 ± 0.07b	4.33 ± 0.21b	0.42 ± 0.57b	6.15 ± 0.02b	0.43 ± 0.07b	3.76 ± 0.2c	0.48 ± 0.58b
	+	SB-L -DSM20194_EPS POS	6.19 ± 0.15a	0.35 ± 0.05b	4.21 ± 0.22b	1.92 ± 0.57a	6.11 ± 0.02b	0.39 ± 0.05b	4.18 ± 0.18b	1.88 ± 0.16a
	-	SB-L -DSM20194_EPS NEG	6.15 ± 0.17a	0.39 ± 0.01b	4.41 ± 0.21b	0.41 ± 0.38b	6.14 ± 0.02b	0.36 ± 0.04b	4.16 ± 0.17b	0.42 ± 0.45b
**400**	-	SB-L_CT	6.18 ± 0.22a	0.25 ± 0.02b	4.89 ± 0.02a	0.33 ± 0.39b	6.08 ± 0.02b	0.26 ± 0.02b	4.39 ± 0.17a	0.14 ± 0.25b
	+	SB-L -B24W	6.12 ± 0.26a	0.26 ± 0.02b	4.12 ± 0.16b	0.31 ± 0.4b	6.1 ± 0.02b	0.24 ± 0.02b	3.71 ± 0.17c	0.29 ± 0.36b
	+	SB-L -SLA5_EPS POS	6.17 ± 0.28a	0.23 ± 0b	4.3 ± 0.14b	0.28 ± 0.2b	6.12 ± 0.02b	0.26 ± 0.02b	3.98 ± 0.14b	0.28 ± 0.44b
	-	SB-L -SLA5_EPS NEG	6.14 ± 0.15a	0.25 ± 0.02b	4.35 ± 0.15b	0.33 ± 0.4b	6.11 ± 0.02b	0.28 ± 0.02b	3.96 ± 0.14b	0.28 ± 0.23b
	+	SB-L -SLA4_EPS POS	6.15 ± 0.16a	0.28 ± 0.03b	4.22 ± 0b	0.32 ± 0.18b	6.16 ± 0.02b	0.27 ± 0.03b	4.02 ± 0.14b	0.31 ± 0.12b
	-	SB-L -SLA4_EPS NEG	6.18 ± 0.01a	0.29 ± 0.04b	4.17 ± 0.14b	0.31 ± 0.43b	6.12 ± 0.02b	0.29 ± 0.04b	3.99 ± 0.14b	0.36 ± 0.44b
	+	SB-L -DSM20193_EPS POS	6.1 ± 0.15a	0.22 ± 0b	4.33 ± 0.12b	1.36 ± 0.04a	6.14 ± 0.02b	0.23 ± 0b	4.1 ± 0.14b	1.43 ± 0.15a

	-	SB-L - DSM20193_EPS NEG	6.19 ± 0a	0.26 ± 0.04b	4.36 ± 0.12b	0.96 ± 0.01b	6.13 ± 0.02b	0.25 ± 0.05b	4.06 ± 0.15b	0.28 ± 0.31b
	+	SB-L - DSM20194_EPS POS	6.21 ± 0.16a	0.27 ± 0.04b	4.4 ± 0b	0.92 ± 0.13b	6.11 ± 0.02b	0.28 ± 0.04b	4.09 ± 0.16b	1.25 ± 0.31a
	-	SB-L - DSM20194_EPS NEG	6.17 ± 0.15a	0.25 ± 0.03b	4.37 ± 0.12b	0.27 ± 0.15b	6.13 ± 0.02b	0.26 ± 0.04b	3.97 ± 0.17b	0.31 ± 0.05b
**450**	-	SB-L_CT	6.16 ± 0.15a	0.24 ± 0.03b	4.71 ± 0.12a	0.25 ± 0.42b	6.14 ± 0.02b	0.26 ± 0.04b	4.41 ± 0.17a	0.34 ± 0.06b
	+	SB-L -B24W	6.08 ± 0.03a	0.25 ± 0b	4.3 ± 0.21b	0.27 ± 0.11b	6.12 ± 0.03b	0.25 ± 0b	3.9 ± 0.23b	0.38 ± 0b
	+	SB-L -SLA5_EPS POS	6.08 ± 0.15a	0.21 ± 0.02b	4.49 ± 0.2b	0.23 ± 0.16b	6.09 ± 0.03b	0.23 ± 0.03b	3.98 ± 0.23b	0.33 ± 0.04b
	-	SB-L -SLA5_EPS NEG	6.05 ± 0.15a	0.19 ± 0.03b	4.47 ± 0.21b	0.22 ± 0.41b	6.1 ± 0.03b	0.17 ± 0.03c	3.96 ± 0.24b	0.23 ± 0.03b
	+	SB-L -SLA4_EPS POS	6.13 ± 0.03a	0.17 ± 0.01c	4.3 ± 0.21b	0.97 ± 0.02b	6.16 ± 0.03b	0.16 ± 0.03c	3.99 ± 0.24b	0.26 ± 0b
	-	SB-L -SLA4_EPS NEG	6.12 ± 0.01a	0.15 ± 0.01c	4.39 ± 0.2b	0.18 ± 0.42b	6.13 ± 0.02b	0.17 ± 0.03c	4.03 ± 0.24b	0.21 ± 0.04b
	+	SB-L - DSM20193_EPS POS	6.1 ± 0.05a	0.19 ± 0.04b	4.46 ± 0b	1.24 ± 0.42a	6.12 ± 0.03b	0.18 ± 0.04b	3.89 ± 0.24b	0.28 ± 0.04b
	-	SB-L - DSM20193_EPS NEG	6.09 ± 0.16a	0.21 ± 0.04b	4.37 ± 0.2b	0.23 ± 0.6b	6.1 ± 0.03b	0.22 ± 0.04b	3.87 ± 0.23b	0.25 ± 0.38b
	+	SB-L - DSM20194_EPS POS	6.11 ± 0.18a	0.23 ± 0b	4.43 ± 0.2b	0.91 ± 0.08b	6.08 ± 0.03b	0.25 ± 0.04b	3.78 ± 0.13c	0.27 ± 0.02b
	-	SB-L - DSM20194_EPS NEG	6.14 ± 0.19a	0.17 ± 0.02c	4.38 ± 0.2b	0.19 ± 0.19b	6.1 ± 0.02b	0.19 ± 0.02b	3.86 ± 0.22b	0.22 ± 0.38b
**500**	-	SB-L_CT	6.13 ± 0.21a	0.19 ± 0.02b	5.02 ± 0.19a	0.21 ± 0.14b	6.15 ± 0.02b	0.18 ± 0.02b	4.62 ± 0.22a	0.21 ± 0.18b
	+	SB-L -B24W	6.11 ± 0.22a	0.16 ± 0c	4.41 ± 0.09b	0.19 ± 0.17b	6.12 ± 0.02b	0.17 ± 0.02c	3.9 ± 0.08b	0.23 ± 0.4b

+	SB-L -SLA5_EPS POS	6.1 ± 0.21a	0.14 ± 0.02c	4.62 ± 0.1a	0.17 ± 0.48b	6.16 ± 0.02b	0.15 ± 0.02c	4.11 ± 0.11b	0.18 ± 0.13b
-	SB-L -SLA5_EPS NEG	6.09 ± 0.22a	0.13 ± 0.02c	4.42 ± 0.09b	0.15 ± 0.9b	6.14 ± 0.02b	0.14 ± 0c	4.06 ± 0.11b	0.17 ± 0.46b
+	SB-L -SLA4_EPS POS	6.12 ± 0.06a	0.15 ± 0.02c	4.42 ± 0.09b	0.97 ± 0.33b	6.13 ± 0b	0.16 ± 0c	4.1 ± 0.01b	0.19 ± 0.49b
-	SB-L -SLA4_EPS NEG	6.13 ± 0.08a	0.12 ± 0.01c	4.35 ± 0.1b	0.18 ± 0.53b	6.15 ± 0.03b	0.13 ± 0c	4.07 ± 0.12b	0.16 ± 0.14b
+	SB-L -DSM20193_EPS POS	6.11 ± 0.15a	0.14 ± 0c	4.6 ± 0.07a	1.32 ± 0.56a	6.12 ± 0.03b	0.15 ± 0.02c	4.02 ± 0.13b	1.39 ± 0.6a
-	SB-L -DSM20193_EPS NEG	6.08 ± 0.18a	0.11 ± 0.03c	4.57 ± 0.06b	0.16 ± 0.41b	6.17 ± 0.03b	0.13 ± 0.03c	4.09 ± 0.15b	0.18 ± 0.03b
+	SB-L -DSM20194_EPS POS	6.14 ± 0.15a	0.15 ± 0.01c	4.46 ± 0.01b	0.9 ± 0.49b	6.1 ± 0.02b	0.17 ± 0.01c	3.79 ± 0.12c	0.19 ± 0.04b
-	SB-L -DSM20194_EPS NEG	6.06 ± 0.15a	0.16 ± 0.01c	4.48 ± 0.06b	0.21 ± 0.22b	6.13 ± 0.01b	0.18 ± 0.04b	3.96 ± 0.15b	0.24 ± 0.18b

[1]D.W., dough weight. Data are mean values of triplicate determination ± standard deviation. a-c means within a column with different letters are significantly different (P <0.05)
